# Quantitative thermophoretic study of disease-related protein aggregates

**DOI:** 10.1038/srep22829

**Published:** 2016-03-17

**Authors:** Manuel Wolff, Judith J. Mittag, Therese W. Herling, Erwin De Genst, Christopher M. Dobson, Tuomas P. J. Knowles, Dieter Braun, Alexander K. Buell

**Affiliations:** 1 Systems Biophysics, Physics Department, Nanosystems Initiative Munich and Center for NanoScience, Ludwig-Maximilians-Universität München, Amalienstr. 54, 80799 München, Germany; 2 Faculty of Physics and Center for Nanoscience (CeNS), Ludwig Maximilians University, Geschwister-Scholl-Platz 1, 80539 Munich, Germany; 3 Department of Chemistry, University of Cambridge, Lensfield Road, Cambridge CB2 1EW, UK

## Abstract

Amyloid fibrils are a hallmark of a range of neurodegenerative disorders, including Alzheimer’s and Parkinson’s diseases. A detailed understanding of the physico-chemical properties of the different aggregated forms of proteins, and of their interactions with other compounds of diagnostic or therapeutic interest, is crucial for devising effective strategies against such diseases. Protein aggregates are situated at the boundary between soluble and insoluble structures, and are challenging to study because classical biophysical techniques, such as scattering, spectroscopic and calorimetric methods, are not well adapted for their study. Here we present a detailed characterization of the thermophoretic behavior of different forms of the protein *α*-synuclein, whose aggregation is associated with Parkinson’s disease. Thermophoresis is the directed net diffusional flux of molecules and colloidal particles in a temperature gradient. Because of their low volume requirements and rapidity, analytical methods based on this effect have considerable potential for high throughput screening for drug discovery. In this paper we rationalize and describe in quantitative terms the thermophoretic behavior of monomeric, oligomeric and fibrillar forms of *α*-synuclein. Furthermore, we demonstrate that microscale thermophoresis (MST) is a valuable method for screening for ligands and binding partners of even such highly challenging samples as supramolecular protein aggregates.

Protein aggregation into highly ordered, insoluble amyloid fibrils and their oligomeric precursors is a hallmark of a range of disorders, many of them neurodegenerative in nature, such as Alzheimer’s and Parkinson’s diseases^1^. In the latter condition, intracellular amyloid deposits, known as Lewy bodies, of the intrinsically disordered protein *α*-synuclein form a major characteristic of the pathology^2^. To date, no cure for this disease exists, a consequence at least in part of the lack of fundamental understanding of the mechanism of aggregation and its associated toxicity, as well as the incomplete characterization of the interactions between aggregates of *α*-synuclein and other compounds, including small molecules and proteins.

Such interactions are important for both diagnostic (e.g. for positron emission tomography^3^) and therapeutic purposes (e.g. for targeted aggregation inhibitors^4^). In this context there is an urgent need for experimental techniques that can be used for high throughput screening to identify such compounds. Standard techniques, such as isothermal titration calorimetry (ITC)^5^ or surface plasmon resonance (SPR)^6^ can provide important information, but suffer from a number of limitations, including high levels of sample consumption (ITC), potential surface artifacts (SPR) and high sensitivity to solution conditions (both ITC and SPR).

Analytical methods based on thermophoresis have recently been introduced as alternatives to these established methods for the measurement of binding interactions of biomolecular compounds^7^,^8^. Thermophoresis, also known as the Soret effect^9^, corresponds to the directed net diffusional flux of particles under the influence of a temperature gradient. If the temperature gradient is stationary, the molecular concentration eventually reaches a steady state through the simultaneous and opposite effects of thermal diffusion (with coefficient D_*T*_) and standard (Fick) diffusion (with coefficient D). The phenomenon of thermophoresis was first described in the 19th century^9^,^10^ and has recently seen a surge in attention, due to its many potential biophysical applications^11^ and even possible role in the origins of life^12^.

In a thermophoretic experiment, the concentration of thermally diffusing particles or molecules, c, can be described by a combination of local equilibrium and non-equilibrium effects, and follows an exponential distribution: 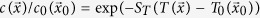
13, where S_*T*_ = D_*T*_/D is the Soret coefficient. Through the creation of well-defined temperature gradients and the subsequent measurement of concentration distributions at steady state, the intrinsic propensities of particles to exhibit thermophoresis can be determined. Temperature gradients can be created through Joule heating^14^, the generation of a hot reservoir^15^ or by absorption of infrared (IR) laser radiation (Fig. 1c)^16^, and the concentration profiles of the species undergoing thermophoresis can be mapped via measurements of variations in refractive index^17^, light scattering^18^ or fluorescence intensity^16^, provided that the species are suitably labeled or show intrinsic fluorescence^11^.

The increasing recognition of the potential importance of thermophoresis for the characterization of biomolecular binding equilibria is paralleled by extensive fundamental research activity on the thermophoretic properties of polymeric and colloidal systems^16^,^19^,^20^,^21^, as well as solvent mixtures^22^. Despite the current lack of an overarching theory of thermophoresis of different systems, interesting trends have been observed in a variety of systems. For nonionic polymers a saturation of the thermal diffusion coefficient after several Kuhn segments has been found in a large set of nonpolar solvents^23^ as well as in water^20^,^24^. Nevertheless, no general tendency of increasing or decreasing Soret coefficient with size for uncharged polymers has been observed^20^,^25^ and theoretical models are still under debate^26^,^27^. For charged polymers, substantial progress has been made in the understanding of ionic effects in recent years. Thermal gradients lead to the development of concentration gradients for the ionic species^28^, which contribute to the movement of charged polymers by the build-up of electric fields^16^,^29^ and diffusiophoresis^30^. Furthermore, thermal gradients also introduce contributions to the Soret coefficient which arise from the change in free energy of the Debye layer associated with the temperature change^31^ and can be described by considering local equilibrium^13^. These models have been successfully tested for spherical particles by variation of ionic strength^16^,^32^,^33^ and extended to elongated structures, such as viruses^34^ and DNA^35^. The Soret coefficients of proteins and various other charged polymers have also been found to increase with temperature in a manner that is described by an empirical formula^17^. Although fundamental research into the origin of this temperature dependence is ongoing, the Soret effect has already been exploited for particle separation^15^ and the detection of phase transitions^36^.

The binding of a ligand to a biomolecule can in many cases induce a change in thermophoretic behavior that is sufficiently large to be detected, and hence a binding curve can be obtained through measurements of a dilution series of one of the binding partners. It has been shown, for example, that binding constants for protein-ligand interactions can be obtained rapidly in this way, even under the most challenging solution conditions and using only minute quantities of sample^8^.

Despite the increasing attention being focused on such effects and the great potential of thermophoresis for high throughput screening, it is not yet possible to predict from first principles the value of the Soret coefficient of any protein under a given set of conditions, or even the sign and magnitude of a change in the Soret coefficient induced by the binding of a ligand. Indeed, very few studies have so far addressed the problem of quantitative measurements of the thermophoresis of proteins^17^ or protein assemblies^37^. The aim of the studies described here is to advance our fundamental understanding of protein thermophoresis through the study of distinct forms of the protein *α*-synuclein. We have chosen this protein because of its relevance to Parkinson’s disease, as well as its well-established ability to form different types of stable aggregates, such as oligomeric structures^38^ and mature amyloid fibrils^39^. In addition, the monomeric protein is kinetically highly stable in bulk solution, and in the absence of catalytic surfaces it does not aggregate at a detectable rate even at high concentrations^40^,^41^, facilitating its study by biophysical techniques. Therefore, *α*-synuclein represents an excellent system through which to study the influence of the size and nature of protein assemblies on their thermophoretic behavior. In particular, we have used a combination of fluorescence correlation spectroscopy (FCS)^42^ and microfluidic free flow electrophoresis^43^, along with measurements of the Soret coefficients of fluorescently labeled monomeric and aggregated *α*-synuclein to examine the importance of electrostatic effects in protein thermophoresis. We find that while the different aggregated species cannot be discriminated based on their electrophoretic mobilities, they exhibit very distinct thermophoretic mobilities. In addition, we show that the binding of a high affinity single domain antibody (nanobody) as well as of a natural small molecule, epigallocatechin gallate (EGCG^44^,^45^) to *α*-synuclein aggregates can be probed by exploiting changes in thermophoretic behavior upon binding. These results establish thermophoresis as a useful method for binding studies to a highly challenging class of target structures.

## Results

### Thermophoresis of protein structures is size-dependent

**Figure 1:**
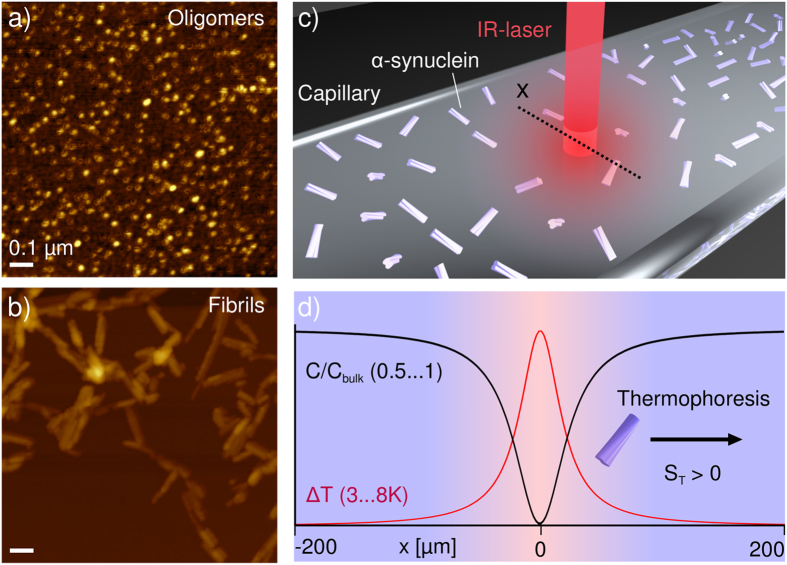
Quantitative thermophoresis of proteins. AFM images of oligomeric (**a**) and fibrillar (**b**) forms of the protein *α*-synuclein, associated with Parkinson’s disease. The oligomers have an average diameter of 15 nm in solution, as determined by fluorescence correlation spectroscopy. (**c**) An initially homogeneous solution, or a suspension of a protein species, here illustrated with fibrillar aggregates, is subjected to localized heating by an IR-laser inside a borosilicate capillary, which leads to directed movement of molecules and complexes along the temperature gradient, until a steady state is established. (**d**) The radially averaged temperature profile (typically established within less than a second after turning on the laser), and the concentration profile (usually established within seconds to minutes) are illustrated schematically at steady state. A schematic fibril is displayed undergoing positive thermophoresis, i.e. migrating away from the heated spot.

**Figure 2:**
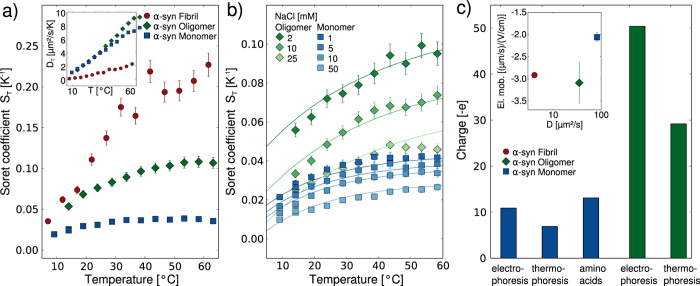
Thermophoretic characterization of three distinct *α*-synuclein species. (**a**) The Soret coefficients, *S*_*T*_, of monomeric, oligomeric and fibrillar *α*-synuclein (in 1 mM Tris buffer at pH 7.4) as a function of temperature, showing their strong size-dependence. Inset: The thermal diffusion coefficient, *D*_*T*_ = *DS*_*T*_, as a function of temperature. (**b**) Fit of the temperature dependence of *S*_*T*_ of *α*-synuclein monomers (blue) and oligomers (green) at different concentrations of added NaCl. The data are globally fitted to a model that includes the electrostatic effects relevant for thermophoresis and where the effective charges of the species and the Soret coefficient of the Tris ion are the only free parameters. (**c**) The charges determined from the fits in (**b**) compared with the charges determined from an analysis of the electrophoretic mobilities (supplementary section 6). For the monomer, the charge expected from the amino acid composition is also plotted. Inset: The free flow electrophoretic mobilities^43^ of fluorescently labeled monomeric, oligomeric and fibrillar *α*-synuclein (in 5 mM Tris buffer pH 7.4) are plotted against their diffusion coefficients (from FCS measurements^42^, and supplementary section 5).

We have produced fluorescently labeled monomeric, oligomeric and fibrillar *α*-synuclein (see supplementary section 2 for detailed protocols) and characterized these different species by atomic force microscopy (AFM, Fig. 1) and FCS (supplementary section 5). For the aggregated forms of the protein, we used a minimally invasive labeling strategy, in which only a small fraction of the protein molecules within each aggregate is labeled. We then measured the Soret coefficients, S_*T*_, of these three distinct and well-defined forms of *α*-synuclein at low ionic strength (1 mM Tris buffer) as a function of temperature (Fig. 2a), using a thermophoresis setup with laser heating and a camera^16^ (see supplementary section 4) to record the time evolution and steady-state distribution of the concentration of fluorescently labeled protein aggregates. In these experiments unlabeled protein molecules and aggregates are invisible. The absolute magnitude of *S*_*T*_ was found to increase with the size of the *α*-synuclein structure (Fig. 2a). A size dependence of the Soret coefficient has been observed previously^13^,^21^,^46^, but the question of whether or not the thermal diffusion coefficient *D*_*T*_ = *DS*_*T*_ also depends on size has been controversial, although most results point towards the size independence of *D*_*T*_ for simple colloid systems^24^,^46^. We find here that while monomeric and oligomeric forms of *α*-synuclein have very similar thermal diffusion coefficients, *D*_*T*_ is markedly smaller for the fibrillar form of the protein (Inset to Fig. 2a). In order to investigate the origin of the size dependence of S_*T*_ in more detail, therefore, we have performed experiments under a range of different solution conditions.

### The thermophoresis of proteins is dominated by electrostatic effects

We first explored the effects of variations in ionic strength on the thermophoretic behavior of the various proteinacious species studied here, as significant effects have been observed previously for DNA^16^. The magnitude of the Soret coefficient decreases when the ionic strength is increased for monomeric and oligomeric *α*-synuclein species (Fig. 2b). The absolute change in S_*T*_ for a given variation in ionic strength is, however, observed to depend on the size and charge of the *α*-synuclein species, with a more pronounced dependence being observed for the oligomers. Similar experiments for fibrillar *α*-synuclein were not pursued because an increase in ionic strength can induce higher order assembly of fibrils, as reported previously^39^, making an accurate determination of the absolute Soret coefficient difficult.

We have recently presented a theoretical description that for DNA quantitatively captures the various electrostatic effects important in thermophoresis, such as the capacitor effect and the Seebeck effect^16^ and (see supplementary section 7). The most important parameters in this model are the size, charge and electrophoretic mobility of the macromolecule under investigation. We have here been able to determine all of these parameters independently for the three distinct types of *α*-synuclein species (inset to Fig. 2c) by using FCS^42^ to measure the standard (Fick) diffusion coefficients (supplementary section 5); the latter can be used to determine the dimensions of the structures. Assuming spherical geometry^38^, we obtain hydrodynamic diameters of 5.6 nm and 15 nm for the monomeric and oligomeric *α*-synuclein, respectively. In addition, the (sonicated) fibril length distributions were characterized in detail by AFM and we found an average length of ~200 nm and a diameter of ~8 nm. Furthermore, we used microfluidic free flow electrophoresis^43^ and (supplementary section 6) to determine the electrophoretic mobility of each species (inset to Fig. 2c). We have fitted the data to a model that takes both the capacitor and Seebeck effects into account (supplementary section 7), allowing us to decompose the Soret coefficient into charge-dependent effects and non-ionic contributions. The fits yield an effective charge that appears to be responsible for the strong dependence of the thermophoresis on the ionic strength of the solution. We can also estimate the charges of the distinct *α*-synuclein species from their electrophoretic mobilities. To that end, the monomeric and oligomeric forms of *α*-synuclein were approximated as spheres and the fibrils as rods, enabling us to use the theoretical framework already developed for colloids^47^, yielding charges of −10.9 e for the monomer, −50.4 e for the oligomer and a value in the range from −200 e to −300 e for the fibrils (supplementary section 6). The value for the monomer is in good agreement with that calculated from the amino acid composition at this pH (−9.1 e) in addition to the charges carried by the fluorescent label (−4 e). It is interesting to see that the oligomers, despite being composed of ca. 30 monomers on average^38^, have a net charge only about 5 times higher than that of the monomer. This difference between expected and determined charge, which is even more pronounced for fibrillar *α*-synuclein, can be explained through a shift of the pK_*a*_ values of the ionizable residues in the aggregates with respect to the monomeric state, as well as the absorption and incorporation of counter ions into the oligomers and fibrils^48^. The values of the effective charges (see Fig. 2c for an overview) of the *α*-synuclein monomers and oligomers calculated from the thermophoretic data (−6.9 e and −29.2 e) are significantly smaller than those resulting from the fits to the electrophoretic mobilities (−10.9 e and −50.4 e). Due to the lack of experimental data on the ionic strength dependence of the S_*T*_ values of the fibrils, we cannot estimate the thermophoretic charge of the fibrils. Since studies that directly compare effective charges determined from electrophoretic and thermophoretic measurements are rare^30^,^33^, the data shown here provide an important benchmark through which to improve the theoretical descriptions of both electrophoretic and thermophoretic phenomena of complex biomolecular structures such as protein molecules and supramolecular protein aggregates. Note that the Soret coefficient of the positively charged Tris ion was determined from a global fit to the ionic strength dependence of the thermophoresis of monomeric and oligomeric *α*-synuclein to be 0.0031/K, and the value obtained is at least comparable in magnitude to the one of the sodium ion with 0.00469/K^16^,^49^.

**Figure 3:**
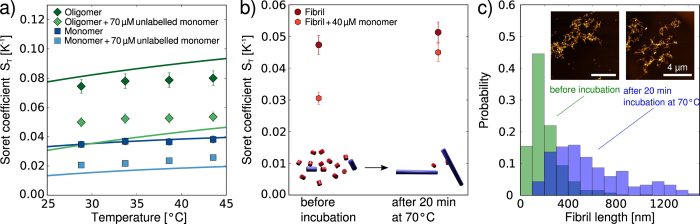
The effect of unlabeled (and hence invisible) monomeric *α*-synuclein on the thermophoresis of the distinct *α*-synuclein species. (**a**) Temperature dependence of S_*T*_ for labeled monomeric and oligomeric *α*-synuclein in the presence and absence of a high background concentration (70 μM) of unlabeled monomeric *α*-synuclein in 1 mM Tris buffer pH 7.4. The solid lines are predictions if the unlabeled monomer is treated as an ionic species within the theoretical thermophoretic model used here (see main text and supplementary section 7). (**b**) The Soret coefficient of fibrillar *α*-synuclein at 20 °C, in the presence and absence of 40 μM unlabeled monomeric *α*-synuclein, before and after a 20 min period of heating to 70 °C. (**c**) Length distributions of the *α*-synuclein fibrils with added unlabeled monomer before and after 20 min heating to 70 °C.

We next tested whether or not the presence of an excess of unlabeled monomeric *α*-synuclein leads to a change in the thermophoresis of the fluorescently labeled monomeric, oligomeric and fibrillar *α*-synuclein (Fig. 3). We find that the thermophoresis of both labeled monomeric and oligomeric *α*-synuclein is decreased by the presence of an excess (70 μM) of unlabeled monomeric *α*-synuclein (Fig. 3a). Furthermore, we find that the Soret coefficient of fibrillar *α*-synuclein at 20 °C in the presence of 40 μM unlabeled monomeric *α*-synuclein shows also a decreased value compared to the sample without added monomeric protein (Fig. 3b). Under these conditions of low ionic strength and relatively low temperature, the rate of incorporation of monomeric protein into the amyloid fibrils is negligibly slow^39^. However, when we heat the samples for 20 min to 70 °C, and again determine the Soret coefficient (Fig. 3b), we find that S_*T*_ has significantly increased for the sample with the added monomer, whereas the increase is smaller for the sample without any added monomer. Analysis of the length distributions of the amyloid fibrils before and after the incubation at 70 °C illustrates that the fibrils have increased in length due to monomer incorporation (Fig. 3c). As the temperature is increased, the structural rearrangements and/or desolvation necessary for the incorporation reaction of the *α*-synuclein monomers into the fibrils are significantly accelerated^39^,^50^.

These results suggest that the presence of unlabeled monomeric *α*-synuclein does indeed affect the thermophoresis of monomeric and aggregated *α*-synuclein. In order to elucidate the physical origin of this effect, we have computed the predicted decrease in Soret coefficient due to the additional Seebeck and capacitor effects caused by the presence of the unlabeled monomer, treating the latter as an additional salt species with the Soret coefficient and charge as determined above for labeled monomer (mean value over the examined temperature range: 
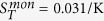
). The predictions are shown in Fig. 3a as solid lines and show that the decrease in Soret coefficient induced by the presence of the unlabeled monomer can be quantitatively described by treating the unlabeled *α*-synuclein monomer as a dissolved electrolyte. It is surprising that this simple approximation yields a relatively good description of the observed behavior, given the limited validity of Debye Hückel theory when treating highly charged macromolecules, such as the *α*-synuclein monomers, as counterions in the capacitor effect. Indeed, the Debye length at the ionic strength that can be formally attributed to the presence of the 70 μM unlabeled *α*-synuclein (ca. 5 nm) is much shorter than the average distance between the protein molecules at this concentration (ca. 30 nm). Nevertheless, based on the agreement between the experiments and the modeling, the decrease in Soret coefficient of the three *α*-synuclein species in the presence of an excess of unlabeled monomeric *α*-synuclein appears to be caused, at least in part, by the electrostatic effects exerted by the latter on the former. In the case of labeled *α*-synuclein monomer, the addition of unlabeled monomer is of course equivalent to an increase in the total concentration of the protein, and it has been reported previously that an increase in concentration of a charged species decreases the Soret coefficient^16^. Since the concentration of unlabeled monomer is far below the overlap concentration (~0.01 Mol/l), effects of the added species upon viscosity are expected to play a minor role here. Furthermore, even at higher viscosities, the steady state distributions of molecules in a temperature gradient are not necessarily affected, due to the dependence of both D and D_*T*_ on viscosity^23^, which can lead to viscosity independent Soret coefficients S_*T*_. Therefore the results of our particular experimental design where only part of the protein molecules are visible allow us to conclude that the effect of an increase in concentration on thermophoresis can be understood in the general framework of electrostatic interactions in the dilute regime.

### Measurement of ligand binding constants to monomeric and aggregated *α*-synuclein

**Figure 4:**
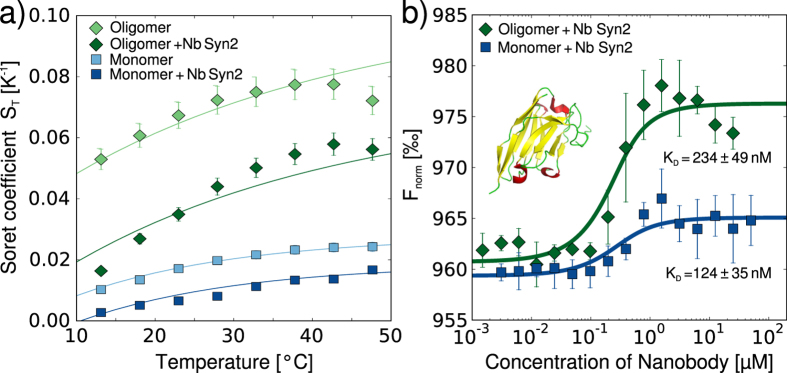
Measurements of the effects of the binding of an antibody on the different *α*-synuclein species. (**a**) Temperature dependence of S_*T*_ for monomeric and oligomeric *α*-synuclein in the presence and absence of the single domain camelid antibody (nanobody) Nb Syn2 (structure of Nb Syn2 with coordinates taken from PDB 2X6M^53^). The solid lines are fits that allow the determination of the reduction in effective charge due to the binding of the nanobody. (**b**) Binding curves of Nb Syn2 to monomeric and oligomeric *α*-synuclein obtained with a thermophoresis setup that monitored the time course of the fluorescence intensity at the position of heating. Each data set combines the results from three independent experiments. The data for the monomer were shifted up by 40‰ for better clarity.

Having established the general principles governing the thermophoresis of protein aggregates, we proceeded to investigate the application of this technique for ligand screening and characterization of the binding of ligands to the aggregates. For this purpose, we investigated the effects of the binding of a single domain camelid antibody (nanobody), which has been shown to bind to the disordered C-terminal region of *α*-synuclein^5^, on the thermophoresis of different *α*-synuclein species. We first determined the S_*T*_ values of monomeric and oligomeric *α*-synuclein in the presence of a saturating concentration of the nanobody (Fig. 4a) and found that in both cases the bound state displays a reduced thermophoretic effect. The nanobody is positively charged at neutral pH (+1.5 e), and therefore the net global charge of the protein-nanobody complex is lower than that of the protein alone. If the observed decreases in Soret coefficient are attributed solely to decreases in charge, it would correspond to a change in charge of +2.7 e for the monomer and +7.0 e for the oligomer (compare solid lines in Fig. 4a). Therefore, at least for the monomer, which is known to bind with a stoichiometry of 1:1 to the nanobody, the observed effect appears larger than expected purely on electrostatic grounds. One reason for this enhanced effect might be the change in overall size and hydrophobicity^51^ associated with the binding of the nanobody. For the oligomers, the value of the reduced charge suggests a stoichiometry much larger than 1:1, which is consistent with the fact that the oligomers consist on average of 30 monomers. However, due to lack of detailed structural information for the oligomer, and hence the accessibility of the binding epitopes, it is difficult to estimate the stoichiometry. We were unable to perform similar experiments with fibrillar *α*-synuclein, as the charge reduction associated with the binding of the nanobody led to almost instantaneous formation of macroscopic assemblies of fibrils, that rendered accurate determination of the Soret coefficient impossible.

Based on the observed change in Soret coefficient upon nanobody binding, however, we tested if the binding constant of the nanobody to both monomeric and oligomeric *α*-synuclein could be measured by using a simplified thermophoresis setup that monitors the time evolution of the total fluorescence intensity in the area of elevated temperature, rather than the full spatial distribution as in the setup used for the determination of the Soret coefficients (integrated vs. spatially resolved approach, see supplementary section 4). In Fig. 4b, we show the corresponding binding curves from the integrated measurements. We obtain binding affinities of the nanobody to monomeric *α*-synuclein that are in excellent agreement with a previously determined value, obtained from ITC measurements (124 ± 35 nM vs. 130 ± 23 nM^5^). The binding affinity to the oligomers (234 ± 49 nM) had not previously been reported, partly due to the challenge of obtaining sufficient quantities of pure oligomers, which we have been able to overcome by exploiting the low sample requirements of thermophoresis. Interestingly, the thermophoresis values of the oligomer-nanobody system in the fully bound state do not reach a stable plateau, but rather decrease linearly. This effect could be caused by the electrostatic influence of the excess free nanobody on the thermophoresis of the oligomer-nanobody complex. In general, electrostatic effects of free ligand molecules might in this way be able to distort binding curves of charged molecules determined by thermophoresis, if the affinity is sufficiently low such that high ligand concentrations need to be employed. However, in the present case, we do not expect a significant influence on the K_*d*_ value, due to the small net charge of the nanobody and the relatively high affinity.

**Figure 5:**
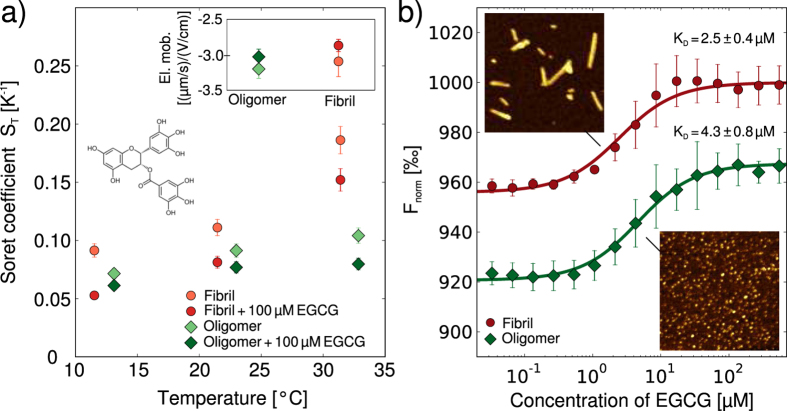
Measurement of binding of EGCG (structure shown in a) to *α*-synuclein aggregates. (**a**) Measurements of the Soret coefficients of oligomeric and fibrillar *α*-synuclein in the presence and absence of 100 μM EGCG. In contrast to thermophoresis, the electrophoretic mobilities remain virtually unchanged in the presence of EGCG (see inset). (**b**) Binding constants for the interactions of EGCG with oligomeric and fibrillar *α*-synuclein were measured using the integrated thermophoresis approach. Each data set is an average of three independent experiments. The insets show AFM images (image sizes are 1 μm × 1 μm) taken after incubation of oligomers and fibrils for 12 h with 100 μM EGCG. No morphological changes could be detected (compare with Fig. 1a,b).

We then investigated the binding of the small molecule epigallocatechin gallate (EGCG), one of the main constituents of green tea, to *α*-synuclein aggregates. This molecule has been reported to bind to various species on the aggregation pathway of *α*-synuclein and even to remodel mature amyloid fibrils^44^. We again first probed whether or not the binding to fibrillar and oligomeric *α*-synuclein manifests itself in a change in Soret coefficient. Despite the fact that EGCG is not charged, we measured a decrease in Soret coefficient upon its binding to both oligomeric and fibrillar *α*-synuclein (Fig. 5a). The global charge of the aggregates is not expected to change upon binding; indeed, the electrophoretic mobilities of the oligomers and fibrils of *α*-synuclein are very similar with and without bound EGCG (see inset of Fig. 5a). Therefore it is likely that changes in the overall protein-solvent interactions are responsible for the observed change in S_*T*_. Indeed, it has been proposed that thermophoresis represents a way of probing interactions of particles and molecules with the solvent^32^. Such a proposition is rendered plausible by a significant, temperature-dependent non-electrostatic contribution to S_*T*_ (see supplementary section 7) that we were able to determine by subtracting the electrostatic contributions from the overall value of S_*T*_.

Furthermore, as in the case of the nanobody, the binding constant of EGCG to oligomeric and fibrillar *α*-synuclein can be determined by using the rapid and straightforward integrated approach (Fig. 5b). In these experiments, we found that the ratio of labeled to unlabeled protein within the aggregates is an important experimental parameter, in particular in the case of a compound that is able to influence the fluorescence intensity of the label upon binding, such as EGCG. In addition, if the ratio of the labeled to the unlabeled protein is too high, the surface properties, and hence the binding behavior of *α*-synuclein aggregates can change significantly as compared to a completely unlabeled structure (supplementary section 4). Using an optimized ratio of labeled to unlabeled protein of ~0.02–0.03 for both aggregate species, we determined the binding constant of EGCG to *α*-synuclein amyloid fibrils and oligomers under these conditions to be 2.5 ± 0.4 μM and 4.3 ± 0.8 μM, respectively. The affinity for the fibrils is approximately one order of magnitude lower than the value reported previously under conditions of higher ionic strength^44^, whereas the affinity of EGCG for oligomers has not previously been measured. It has, however, been reported that EGCG can induce structural changes in amyloid fibrils and other protein aggregates^44^, and such a substantial structural rearrangement can be expected to affect the thermophoretic behavior, complicating the determination of binding constants. In order to test whether or not such effects occurred during our binding studies, we incubated oligomeric and fibrillar *α*-synuclein for 12 h in the presence of 100 μM EGCG followed by AFM imaging, to allow for sufficient time for even slow remodeling processes to take place. The resulting images are shown as insets in Fig. 5b and reveal no noticeable change in morphology compared to freshly prepared oligomers and fibrils (compare with Fig. 1c,d), suggesting that under the conditions employed here, no significant remodeling of fibrils and oligomers is induced by EGCG.

## Discussion

We describe in this paper the results of a comprehensive experimental approach that has allowed substantial progress to be made towards a quantitative understanding of the thermophoresis of monomeric and aggregated forms of proteins. The approach consists of a combination of precise control of sample preparation (solution conditions, labeling position and density), independent and quantitative measurements of size, electrophoretic mobility and thermophoretic mobility, as well as theoretical modeling. With this strategy, we have shown that electrostatic effects, in particular electrophoresis in the field created by the temperature-induced ion gradient (‘Seebeck effect’), as well as the temperature dependence of the ion cloud extension and its associated electrostatic energy (‘capacitor effect’) (supplementary section 7 and for details of the modeling process)^16^, are important factors in the thermophoresis of proteins and protein aggregates. These conclusions are consistent with those reported previously for nucleic acids^16^,^32^, and therefore suggest that there may be universal principles that govern the thermophoresis of charged macromolecules. Such a general understanding of the well-established physico-chemical phenomenon of thermophoresis represents an important step in the further development of this technique as an experimental strategy for characterizing biomolecular interactions.

We were able to combine the advances made using custom built research tools, which allow for the characterization of a range of well-defined physical properties (diffusion coefficient, electrophoretic mobility and Soret coefficient), with the ease of use of a simplified thermophoresis setup that can be applied in a straightforward manner for the determination of binding constants. This dual strategy has allowed us to exploit the precise control of sample preparation for the detection and characterization of the binding of a range of ligands (small molecules and macromolecules) to different types of disease-related protein aggregates. Indeed, we were able to investigate structures ranging from soluble monomeric protein molecules to oligomeric structures and mature amyloid fibrils. Because of the importance of electrostatic effects in defining the magnitude of the observed thermophoretic effect, these results show that thermophoresis is very well suited for the study of binding events that lead to a change in charge.

Even in cases where there is no change in global charge, however, we found that thermophoresis allowed for detection of binding events, due to the additional dependence on parameters such as size and interaction with the solvent. Indeed, we observed that protein aggregates that are very different in size and structure, but which display a very similar electrophoretic mobility, such as oligomeric and fibrillar *α*-synuclein, show a marked difference in thermophoretic mobility. Furthermore, the binding of a ligand that does not lead to a change in global charge and electrophoretic mobility, such as of the compound EGCG, to oligomeric and fibrillar *α*-synuclein leads to a detectable change in thermophoretic behavior. It has recently been shown in this regard that the binding of EGCG to *α*-synuclein oligomers changes their surface properties significantly, and renders them less disruptive to lipid membranes^45^. The thermophoretic response to changes in surface hydrophobicity reflects the physical complexity of systems with non-uniform temperature that leads to a potentially greater discriminatory power compared to other separation techniques based only on size or electrophoretic mobility.

## Conclusions

The results of this study have contributed significantly to the rationalization of the size and charge-dependence of protein thermophoresis, and therefore have significantly advanced our fundamental understanding of this phenomenon and enabled a general strategy to be outlined that would deepen this understanding further in the future. This strategy consists of specifically designed sample preparation and labeling strategies, combined with state-of-the-art thermophoretic, electrophoretic and size measurements of the structures under study and theoretical analysis and modeling of the physico-chemical factors that determine thermophoresis. We have been able to show that species such as oligomeric and fibrillar protein aggregates show very similar electrophoretic, but very distinct thermophoretic behavior.

Equally importantly, we have also established the possibility of using thermophoresis for the screening and characterization of ligand binding to disease-related protein aggregates. Despite the fact that microscale thermophoresis is in the process of becoming a well-established experimental technique for protein-ligand binding affinity measurements, it has not previously been reported for the study of ligand binding to supramolecular aggregates, such as amyloid fibrils. Due to their polymeric nature these types of samples are substantially more challenging to handle than most soluble monomeric proteins and we present here a comprehensive protocol for the use of microscale thermophoresis for amyloid-ligand binding assays. The possibility of characterizing novel types of ligands that bind to protein aggregates, both small and large molecules, at high throughput and using minute sample quantities is a highly valuable addition to the experimental toolbox available for the development of diagnostic and therapeutic strategies against protein misfolding diseases. In particular, we would like to emphasize that oligomeric structures, which have been highlighted as the most toxic species on the aggregation pathway^52^, often occur only at low concentration and with short lifetimes and hence experimental methods that are rapid and require only small amounts of sample are vital for their study.

## Materials and Methods

Detailed protocols for sample preparation and for the measurements of diffusion coefficients, electrophoretic mobilities, Soret coefficients and binding curves can be found in the supplementary materials.

## Additional Information

**How to cite this article**: Wolff, M. *et al*. Quantitative thermophoretic study of disease-related protein aggregates. *Sci. Rep*. **6**, 22829; doi: 10.1038/srep22829 (2016).

## Supplementary Material

Supplementary Information
