# Supplementary material for: Quantitative thermophoretic study of disease-related protein aggregates
Source: Sci Rep. 2016 Mar 17;6:22829. doi: 10.1038/srep22829 (PMC4794802; doi:10.1038/srep22829)
Supplement: Supplementary Information [file srep22829-s1.pdf]

Supplementary information: Quantitative thermophoretic study of disease-related protein aggregates

Manuel Wolff[1], Judith J. Mittag[2], Therese W. Herling[3], Erwin De Genst[3], Christopher M. Dobson[3], Tuomas P.J. Knowles[3], Dieter Braun[1,*] and Alexander K. Buell[3,4,*]

[1]Systems Biophysics, Physics Department, Nanosystems Initiative Munich and Center for NanoScience, Ludwig-Maximilians-Universität München, Amalienstr. 54, 80799 München, Germany
[2]Faculty of Physics and Center for Nanoscience (CeNS), Ludwig Maximilians University, Geschwister-Scholl-Platz 1, 80539 Munich, Germany
[3]Department of Chemistry, University of Cambridge, Lensfield Road, Cambridge CB2 1EW, UK
[4]Present address: Institute of Physical Biology, University of Düsseldorf, Universitätsstr.1, 40225 Düsseldorf, Germany

*authors to whom correspondence should be addressed: dieter.braun@lmu.de, alexander.buell@uni-duesseldorf.de

Content

1. Chemicals and reagents
2. Preparation of protein samples and protein labeling
3. Atomic force microscopy
4. Thermophoresis experiments
5. Fluorescence correlation spectroscopy of monomeric and aggregated α-synuclein
6. Free flow electrophoresis measurements of monomeric and aggregated α-synuclein
7. Modeling the electrostatic effects in protein thermophoresis
8. ComSol simulations of the thermophoresis setup

1 Chemicals and reagents

Thioflavin-T (ThT), Epigallocatechin gallate (EGCG), NaCl, Na_2HPO_4, NaH_2PO_4, PBS tablets and Tris-HCl were purchased from Sigma Aldrich. BCECF, Alexa Fluor® 568 C_5 maleimide and Alexa Fluor® 647 C_2 maleimide were purchased from Life Technologies Ltd (Paisley, UK).

2 Preparation of protein samples and protein labeling

2.1 Monomeric unlabeled and labeled α-synuclein

Wild type α-synuclein (α-syn) was recombinantly expressed and purified as reported previously [1]. After the last step of the purification protocol, the protein solution (in 20 mM phosphate buffer) was divided into aliquots of 500-1000 µl at concentrations between 200 and 300 µM, flash frozen with liquid nitrogen and stored at -80 °C. When the monomeric protein was required in a different buffer, it was dialysed for 24 h against a thousandfold larger volume of the required buffer. For the fluorescent labeling, we used the N122C variant [2], in order to be able to attach the fluorescent label. This variant was expressed and purified similarly to the wild type protein, except that the lysis buffer and all subsequent buffers contained 1 mM EDTA and 1 mM DTT. For the labeling, ca. 0.2 µmol of N122C variant α-syn in 500 µl buffer was injected into a Superdex 200 Increase 10/300 GL gel filtration column (GE Healthcare, Little Chalfont, UK) that had been incubated with labeling buffer (Tris or phosphate buffer at ~pH 7, 5-20 mM). The fluorescent dyes (maleimide derivatives) were dissolved at 10 mg/ml in DMSO or DMF and 100 µl of this stock solution were added to the combined protein fractions immediately after elution. The protein was left to react with the label for 1 h at room temperature and afterwards for 12 h at 4 °C. Then the solution was concentrated to 500 µl

using Amicon centrifugal concentrators with 3 kDa MW cut-off (Millipore, Watford, UK) and injected into a Superdex 200 Increase 10/300 GL gel filtration column that had been incubated with the buffer the protein was required in, i.e. 20 mM PB buffer pH 6.5 or 5 mM Tris buffer pH 7.4. The most concentrated fractions of the labeled protein were combined and divided into aliquots of 25 µl. The aliquots were flash frozen in liquid nitrogen and stored at -80 °C until use. Labeling efficiency was evaluated with mass spectrometry (Figure 1 a) and the protein concentration was determined using amino acid analysis (both services provided by PNAC facility, Department of Biochemistry, University of Cambridge). We also kept some of the fractions of free dye label for control experiments (see below).
The single chain camelid antibody NbSyn2 was expressed and purified as described in [3].

2.2 Preparation of labeled α-synuclein oligomers

The preparation of stable α-synuclein oligomers is similar to the protocol described in detail in [4]. In short, monomeric α-synuclein is dialysed for 1-3 days against pure water. The solution is then freeze-dried and the lyophilized protein is stored at -80 °C until use. For oligomer formation, the dry protein is dissolved in phosphate buffer saline (PBS) at concentrations between 600 and 800 µM and incubated at 37 °C for several hours (10-20 h) under quiescent conditions. In most cases, the protein solution had not visibly aggregated/gelled despite the high protein concentration after this incubation period. It has been shown that quiescent α-synuclein solutions in the absence of pre-formed seeds [5] and of other aggregation stimulating conditions, such as lipid bilayers [6] aggregate only very slowly, due to the fact that the fibril nucleation is a heterogeneous process that requires catalytic interfaces, such as the air-water interface [7]. 500 µl of the protein solution are then injected into a Superdex 200 Increase 10/300 GL gel filtration column, which had been incubated with 5 mM Tris buffer. The protein is eluted at 0.5 ml/min and the oligomers elute as a small peak of ca. 0.75 ml volume after ca. 20 min, followed by a largely dominant monomer peak (Figure 1 b).
In order to produce fluorescently labeled oligomers, we reasoned that it would be best to minimize the labeling density; the lowest possible label density corresponds to one dye molecule per oligomer. Using various light scattering techniques, we have in previous work determined these stable α-synuclein oligomers to consist of $\sim$30 monomers on average [4]. Therefore, we used ratios of unlabeled to labeled protein of 30 or higher. Figure 1 b shows an example of a chromatogram illustrating the relative populations of oligomers and monomers and giving an idea about the labeling density.

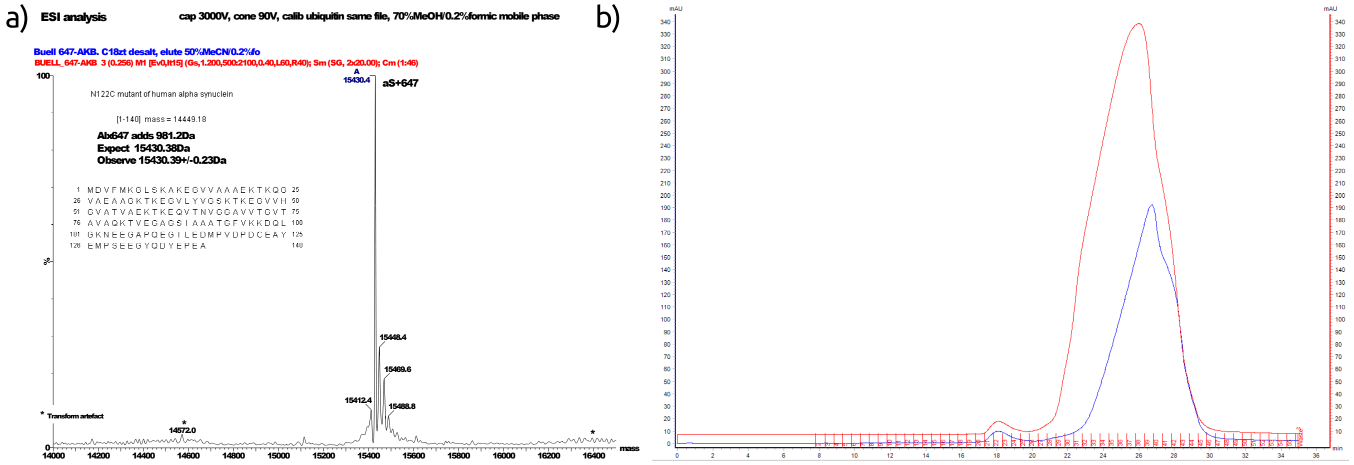

Figure 1: a) Mass spectrum of α-synculein N122C labeled with Alexa Fluor® 647, showing the essentially quantitative labeling. b) Example of a size exclusion chromatogram of a mixture of labeled (with Alexa Fluor® 647) and unlabeled α-synuclein, treated according to the protocol described above. The small peak to the left of the large monomer peak corresponds to the oligomers (red: absorption at 647 nm; blue absorption at 280 nm). Fractions 22 and 23 were combined and used as the oligomeric species. It can be seen that the labeled and unlabeled monomers have slightly different retention times. Also, the relative absorptions at 280 and 647 nm are comparable for the monomer and oligomer peaks, suggesting that the labeled monomer is incorporated statistically into the oligomers and that therefore the labeling density of the oligomers corresponds to the initial proportion of labeled monomer.

Our attempts to label the oligomers proved insightful regarding their mechanism of formation. Adding the labeled protein at different points in the oligomer formation protocol led to very different incorporation efficiencies. When the labeled protein was added after the freeze-dried unlabeled protein had been dissolved in PBS, virtually no labeled oligomers were obtained. Furthermore, the duration of the incubation had very little effect on the labeling yield.

However, the best incorporation efficiency (equal to the ratio of added labeled protein) was achieved when labeled and unlabeled protein were mixed before the dialysis against water. These findings strongly suggest that these stable α-synuclein oligomers form during the process of dialysis and/or lyophilisation and are therefore possibly not directly connected with the molecular pathway that leads to the formation of amyloid fibrils; we have drawn similar conclusions in the past based on kinetic experiments with these oligomers [4].
After gel filtration, the most concentrated oligomer fractions were combined (typically ∼500 µl) and concentrated to about ten times the eluted concentration, using Amicon centrifugal concentrators with 3 kDa MW cut-off (see above). For the measurements of the Soret coefficients, we required the oligomers in 1 mM Tris buffer. In those cases we diluted the concentrated oligomers 1:4 into water and re-concentrated them to the desired final concentration. Once the oligomers were purified and concentrated, they were stable and could be used for several days, as confirmed by fluorescence correlation spectroscopy (see below).

2.3 Preparation of labeled α-synuclein fibrils

Fibrils with varying labeling densities were prepared by seeding a mixture of labeled and unlabeled protein with small percentages (5% or less) of unlabeled fibrils prepared at pH 6.5 and sonicated to create short seeds as described in [5]. For the accurate measurements of S_T, the seeds were incubated with the monomeric fractions (either directly as eluted or concentrated) from the oligomer purification experiments, yielding the same range of labeling densities as for the oligomers ($\leq$ 1:30). Under the conditions of low ionic strength (5 mM Tris buffer, pH 7.4) under which the oligomers were purified, the growth of the seed fibrils is relatively slow, due to electrostatic repulsion between the monomer and the fibril end [8]. Therefore we incubated the seeded protein solution at elevated temperature (50 °C) for 2-3 days under heavy stirring with a magnetic stirrer. We verified by fluorescence correlation spectroscopy (FCS, see below) that the monomer content of these fibril samples was below 20%. Under those conditions of very low ionic strength, that have to our knowledge not been studied in detail before, α-synuclein appears to have a relatively high concentration of free monomer at equilibrium, presumably because the free energy of aggregation has an unfavorable electrostatic component [9] that contributes more strongly at lower ionic strength. The yield can be improved by adding NaCl (10 mM) to the seeded protein solution, which can be tolerated as the subsequent dilution of the fibril sample for the thermophoretic measurement will sufficiently dilute the NaCl. Due to the slow dissociation of monomer from the fibril ends, such a diluted fibril suspension will not immediately re-equilibrate to a high free monomer concentration. For the measurements of the Soret coefficients and for FCS measurements, the fibrils were diluted 30-60 fold (to achieve suitable signal intensity and particle counts) into 1 mM Tris buffer pH 7.4 and sonicated for 5 min with a Sonopuls 2070 sonicator with a MS 72 tip (Bandelin electronic, Berlin, Germany) at minimal power and 30% pulses.
Our overall strategy of the production of labeled fibrils was based on our previous extensive studies of α-synuclein fibril formation and growth [5]. We avoided the production of labeled fibrils at higher values of the ionic strength, as we had previously found that the higher order assembly ('flocculation') of fibrils induced at higher ionic strength values is only partly reversible upon dilution into lower ionc strength buffer, even after sonication. In all cases, we produced labeled fibrils by seeded growth reactions, as we have previously shown that in seeded growth, the kinetics of aggregation is independent of the ratio of labeled to unlabeled monomer [2]. Therefore, it is reasonable to assume that the labeled monomer will be incorporated statistically into the growing fibrils, and that therefore the labeling densities correspond to the initial proportions of labeled to unlabeled monomer.
For the measurements of small molecule binding using the commercially available Monolith instrument (Nanotemper, Munich, Germany), the absolute concentration of the amyloid fibrils is an important parameter, and hence we prepared the fibrils from mixtures of unlabeled and labeled protein solutions at known concentrations. 5% seed fibrils were incubated with a total of 50 µM of monomeric protein with varying proportions of labeled and unlabeled α-synuclein molecules in 20 mM phosphate buffer pH 6.5. The samples were incubated at 37 °C overnight, then they were diluted 1:2 into H_2O, sonicated for 3 s and incubated at room temperature overnight. Then the samples were flash frozen in liquid nitrogen and stored at -80 °C until used. No differences in morphology were observed by AFM between fibrils prepared with different proportions of labeled protein.

3 Atomic force microscopy (AFM)

AFM images of purified oligomers and sonicated fibrils were taken using a Nanowizard II atomic force microscope (JPK, Berlin, Germany) using tapping mode in air. The samples were diluted to ∼1 µM total protein concentration in water and 10 µl were deposited on freshly cleaved mica (Agar Scientific, Stansted, UK) and left to dry. The length distributions were extracted with in-house written Python code, where the ends of the fibrils were manually selected and the program then draws a line onto the fibril, in order to avoid double-counting of fibrils.

4 Thermophoresis experiments

We pursued a dual strategy in this work in order to both improve our fundamental understanding of the physical origins of protein thermophoresis and to demonstrate its usefulness in screening strategies for ligands of disease-related protein aggregates. We performed measurements of the Soret coefficients of different types of aggregates under different conditions in order to test the applicability to proteins of the theoretical description of biomolecular thermophoresis that we have presented in the past for the simpler case of nucleic acids [10, 11]. These experiments were performed using a home-built setup, based on an inverted fluorescence microscope [11], that records the full spatial distribution of fluorescence intensity, and hence concentration of α-synuclein species, as a function of time. Having optimized our experimental protocols (labeling density, solution conditions etc.), we then performed detailed binding experiments with monomeric, oligomeric and fibrillar α-synuclein and small molecule and protein ligands using a commercially available instrument, that measures the total fluorescence intensity in the region of interest as a function of time.
For the measurements of Soret coefficients, the concentration of labeled α-synuclein was between 0.1 and 1 μM. Correspondingly, the total protein concentration (by mass) was 30-50 fold higher in the case of the oligomers and fibrils, as these species were produced from mixtures of unlabeled protein doped with 2-3% fluorescently labeled monomer. For the measurements of the binding constants of the antibody NbSyn2 and the small molecule EGCG to oligomeric and fibrillar aggregates, the total protein concentrations by mass were between 0.2 and 0.5 μM with the concentration of labeled protein accordingly 30-50 times lower. For accurate measurements of binding constants, it is important to keep the concentration of one binding partner constant at a value at or below the expected K_D. The resulting low concentrations of labeled α-synuclein were compatible with the sensitivity of the instrument (see below).

4.1 Measurements of Soret coefficients of monomeric and aggregated α-synuclein

Measurements were performed with an upright fluorescence microscope (Zeiss Vario Scope.A1) using an air objective (Zeiss EC-Plan NeoFluar, 40x, NA=0.9), a CCD camera (Andor Luca DL-658M-TIL) and heating from an infrared laser (Fibotec, λ = 1480 nm absorbed in water) [11], coupled into the optical path right above the objective. To keep convection artifacts below experimental error, measurements were performed in borosilicate capillaries with an inner rectangular cross section of 50 x 500 μm^2 (VitroCom Vitrotubes #5005-050). The thin sample, low numerical aperture and moderate concentration depletion (<50%) ensured that temperature and concentration profiles were equally averaged along the optical axis. For a sketch of the setup, see Figure 2.
The chamber base temperature was controlled by a PID loop using Peltier elements (Telemeter Electronic GmbH, PC-128-10-05) and a heat bath. The chamber height of 50 μm and the moderate temperature rise of less than 9 K above base temperature kept thermal convection small. The measurement was automated and the LED, IR, motorized stage, temperature, and camera trigger were controlled with LabVIEW. The response of the concentration of labeled protein in space and time was recorded at 2.5 Hz by fluorescence imaging. Ten seconds of the equilibrated sample were imaged, followed by different time periods of thermophoretic depletion (depending on the nature of the sample) under optical heating and different time periods to monitor the back-diffusion after switching off the laser.
The profile of the intermittent local optical heating was measured using the temperature dependent fluorescence of the dye BCECF (acid form, Invitrogen B-1151) at a concentration of 50 μM in 10 mM Tris, pH 7.8 (Figure 3a and b). First the peak temperature ΔT for the applied laser power was determined in two dimensions by the assumption of Lorentzian temperature profile $T(r) = T_0 + \Delta T \frac{w^2}{(r^2+w^2)}$. Second the temperature dependence of ΔT as a function of base temperature was assumed as predicted by COMSOL simulations and confirmed by measurements (supplementary section 8). The Soret coefficient was deduced by evaluating the radial concentration profile at steady state in correspondence to the image after temperature jump according to $S_T \Delta T(T, r) = -\log\left(\frac{c(r)}{c_0}\right)$ (Figure 3c and d). The error bars for individual Soret coefficients are based on the uncertainties of the temperature jump ΔT (see also supplementary section 8).

4.2 Measurements of ligand binding affinities of monomeric, oligomeric and fibrillar α-synuclein

The measurements of the ligand binding constants were performed with a Monolith NT.115 instrument (Nanotemper, Munich, Germany). We obtained the best experimental results (complete absence of sample absorption onto the inner walls of the glass capillaries) using the hydrophobically coated glass capillaries provided by Nanotemper.
The binding experiments between monomeric/oligomeric α-synuclein and the nanobody NbSyn2 were performed as follows. A dilution series was prepared in PBS buffer of the Nb (from 202 μM-6.2 nM, 10 μl at each concentration). The labeled monomers (in PB pH 6.5) were diluted to a concentration of 0.4 μM into H_2O, and then 10 μl of the diluted protein solution were added to the 10 μl aliquots of the NbSyn2 dilution series, yielding a final monomer concentration

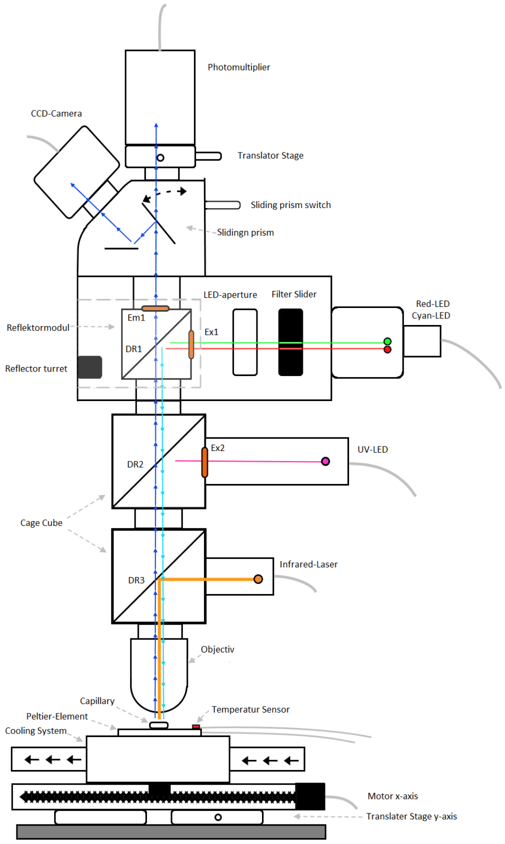

Figure 2: Schematic illustration [12] of the thermophoresis setup for quantitative measurements of the Soret coefficients of protein aggregates. The setup is based on a fluorescence microscope. The heating is carried out with an infrared laser that is coupled into the light path. Detection can be performed either with a CCD camera or with a PMT.

of 0.2 µM. The glass capillaries were filled through capillary action and thermophoretic measurements were performed (25 °C, 15% red LED intensity, 20 and 40% IR laser intensity, 30 s laser on). The labeled oligomers were diluted in 5 mM Tris buffer pH 7.4 to a concentration of approximately 1 µM, as estimated from the size chromatogram during the isolation and purification of the oligomers. Then 10 µl of the oligomer solution were added to the 10 µl of the NbSyn 2 dilution series and the capillaries were filled and thermophoresis measurements performed analogously to the monomer case, except that the LED intensity was 60%. Representative data for nanobody binding to monomeric and oligomeric α-synuclein are shown in Figure 4. In the case of the oligomers, the thermophoretic amplitude was measured before steady state was reached (ca. 5 s after the heating laser was switched on), due to the fact that the data became noisier at later times. This is presumably due to the higher order assembly of the oligomers, that experience a decrease in charge due to the nanobody binding. In the case of the monomer, the thermophoretic amplitude was measured at steady state, 30 s after the heating laser was switched on.

For the experiments with EGCG, we first prepared stock solutions of 50 mg/ml (109.08 mM) in dimethylsulfoxide (DMSO) and then diluted it 50-fold into water to reach a concentration of 2.18 mM with 2% residual DMSO. We then prepared a dilution series into water with 2% DMSO (10 µl per sample) and added 10 µl of the monomer (1.5 µM total monomer, 2% labeled, 98% unlabeled monomer), oligomer or fibril (1.5 µM total protein, 2% labeling density) samples. Then we performed thermophoresis experiments at 25 °C and 20 as well as 40% of the IR laser power.
During our experiments that probed EGCG binding to oligomeric and fibrillar α-synuclein, we noticed that the apparent binding constant changed during the first hour after sample preparation; the binding was observed to become tighter (Figure 5). While we are not certain about the origin of this change in apparent affinity, it is unlikely to be caused by a pronounced change in aggregate structure (see AFM images in Figure 5 of main manuscript), but could indicate multi-step binding with rapid kinetics of initial and slower kinetics of later stages. An alternative explanation could also be a chemical modification of the fluorescent label by the bound ligand. The experiments shown in the main manuscript were performed after 1 h incubation of the samples.
For all the measurements of binding constants, we combined data from three independent experiments.
We also performed binding and stability time course experiments with fibrils prepared with different percentages of

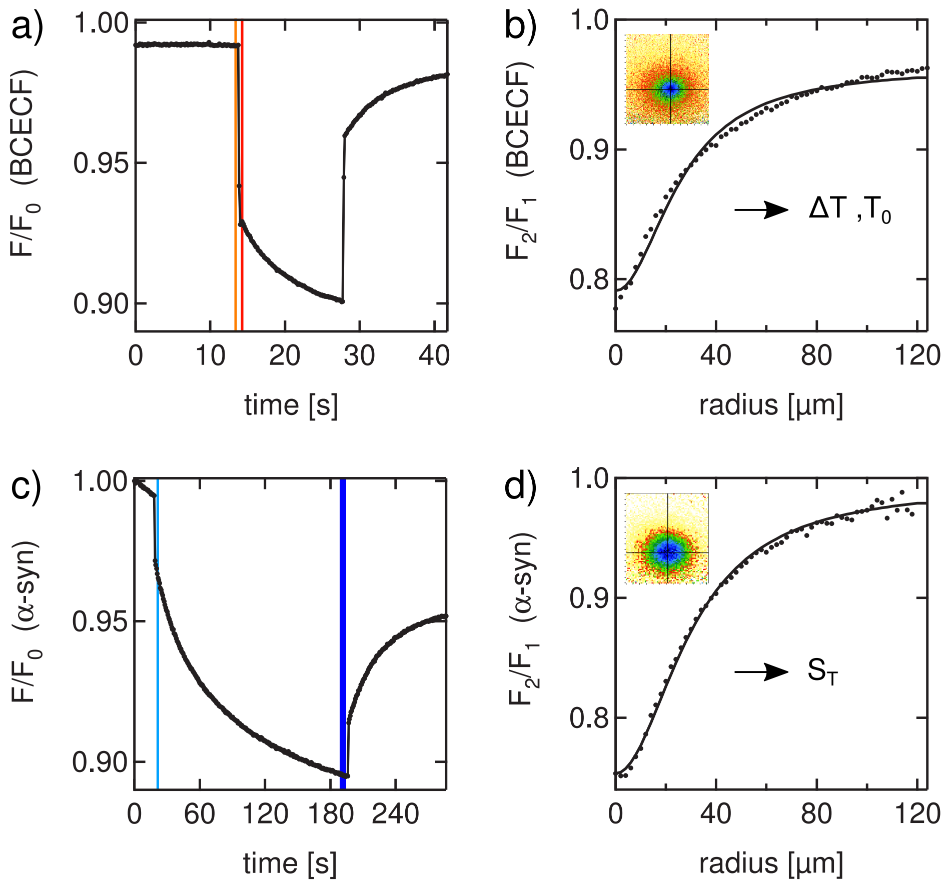

Figure 3: Data evaluation for the quantitative measurements of the Soret coefficient. The temperature gradient which was induced by the IR-laser was determined by BCECF fluorescence. a) The mean fluorescence on the CCD camera drops when the temperature gradient is established within 300 ms. Afterwards thermophoresis takes place. When the IR-laser is turned off the mean fluorescence recovers due to back diffusion. Dividing the picture after the temperature jump (red bar) by the one beforehand (orange bar) gives the radial fluorescence profile as indicated in b). The radial fluorescence profile is approximated by a Lorentzian function and the temperature gradient is then determined with the help of a calibration curve. c) For the α-synuclein species, here illustrated with monomer, the mean fluorescence is monitored over a longer period of time to reach a steady state. The change of concentration due to thermophoresis is obtained by division of the mean picture at the steady state (dark blue bar) by the one after temperature jump (light blue bar). d) The radial concentration profile together with the temperature profile gives the respective Soret coefficient. Here the measurement of α-synuclein monomer at 1 mM Tris pH 7.4 is shown. The base temperature is set to 25 °C in the presented examples.

incorporated labeled protein. Figure 6 a) shows a comparison of experiments with fibrils with 2% and 50% labeling density. It can be seen that the apparent affinity and the time-dependent behavior depend on the labeling density. The overall result of these studies was that the labeling density should be minimized. Obviously, it is always desirable to minimize the impact that any label might have on the process under study, but if the label concentration is decreased too much, then the signal is too weak at the low (µM) total protein concentrations that needed to be used in order to probe the binding affinity accurately. We found that 2% of labeled protein in the fibrils gave consistently the best results.

We also performed oligomer binding experiments with Thioflavin-T instead of EGCG (Figure 6 b), starting with a 2.33 mM ThT solution in H_20 with 2% DMSO. A weak interaction could be detected, that displays no time dependence. Furthermore, we found that also monomeric α-synuclein interacts with EGCG (Figure 6 c), whereas the free dye label shows no change in thermophoresis upon incubation with an EGCG concentration series, confirming that the binding curves presented in this work are not artifacts from ligand-label interactions.

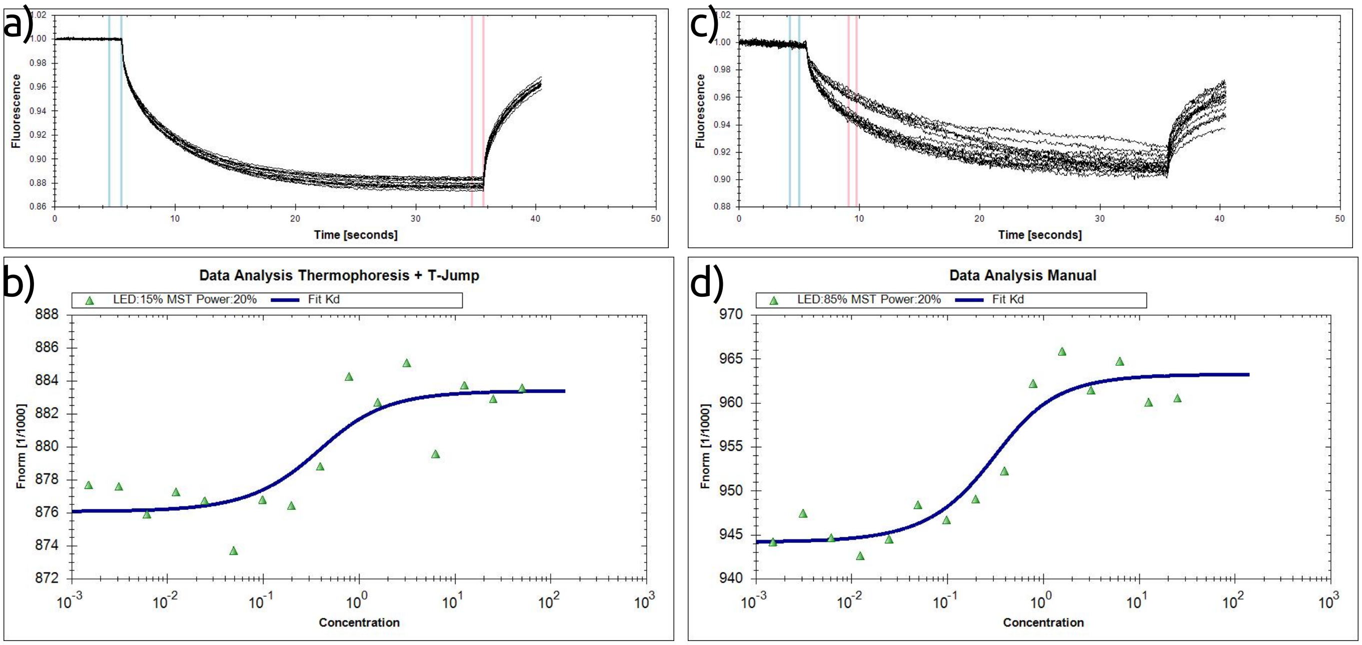

Figure 4: Microscale thermophoresis (MST) binding data of monomeric and oligomeric α-synuclein with the nanobody NbSyn2. a) Raw MST curves for monomeric α-synuclein. b) Binding curve for monomeric α-synuclein. c) Raw MST curves for oligomeric α-synuclein. d) Binding curve for oligomeric α-synuclein.

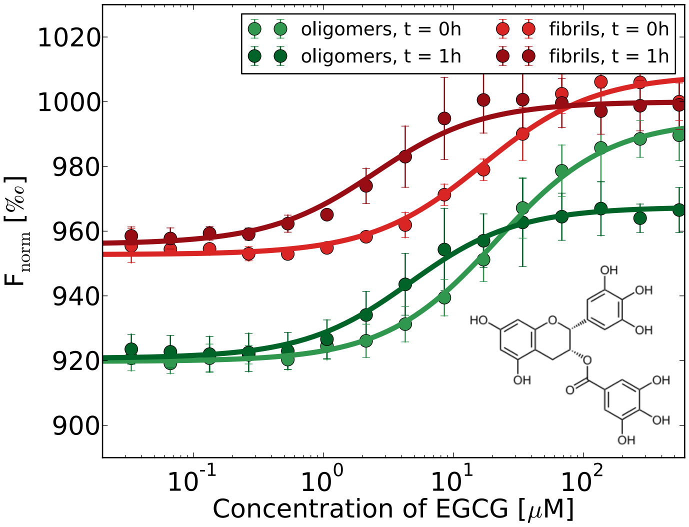

Figure 5: Change in apparent binding affinity of EGCG to oligomeric and fibrillar α-synuclein. For both species, the binding appears to become tighter over the course of an hour, changing from 16.5±1.6 μM and 21.2±2 μM, for fibrils and oligomers, respectively, to 2.5±0.4 μM and 4.3±0.8 μM.

5 Fluorescence correlation spectroscopy (FCS) of monomeric and aggregated α-synuclein

FCS measurements were performed on a Axiovert 200 microscope equipped with a ConfoCor2 unit (Carl Zeiss Jena, Germany), a 543 nm and 633 nm helium-neon laser and an apochromatic 40x water-immersion objective with a NA of 1.2 (Carl Zeiss). Fluorescence emission was separated from laser light using a bandpass filter (560-615 nm) for excitation with 543nm and a long pass filter (650 nm) for excitation with 633 nm. Calibration was performed with Alexa546 or Alexa633, respectively, to determine the dimensions of the observation volume. Samples were filled in NUNC 8-Well-Plates (Thermo Scientific). All measurements were performed at room temperature (22 °C, air conditioned). For analysis the ConfoCor2 software was used.

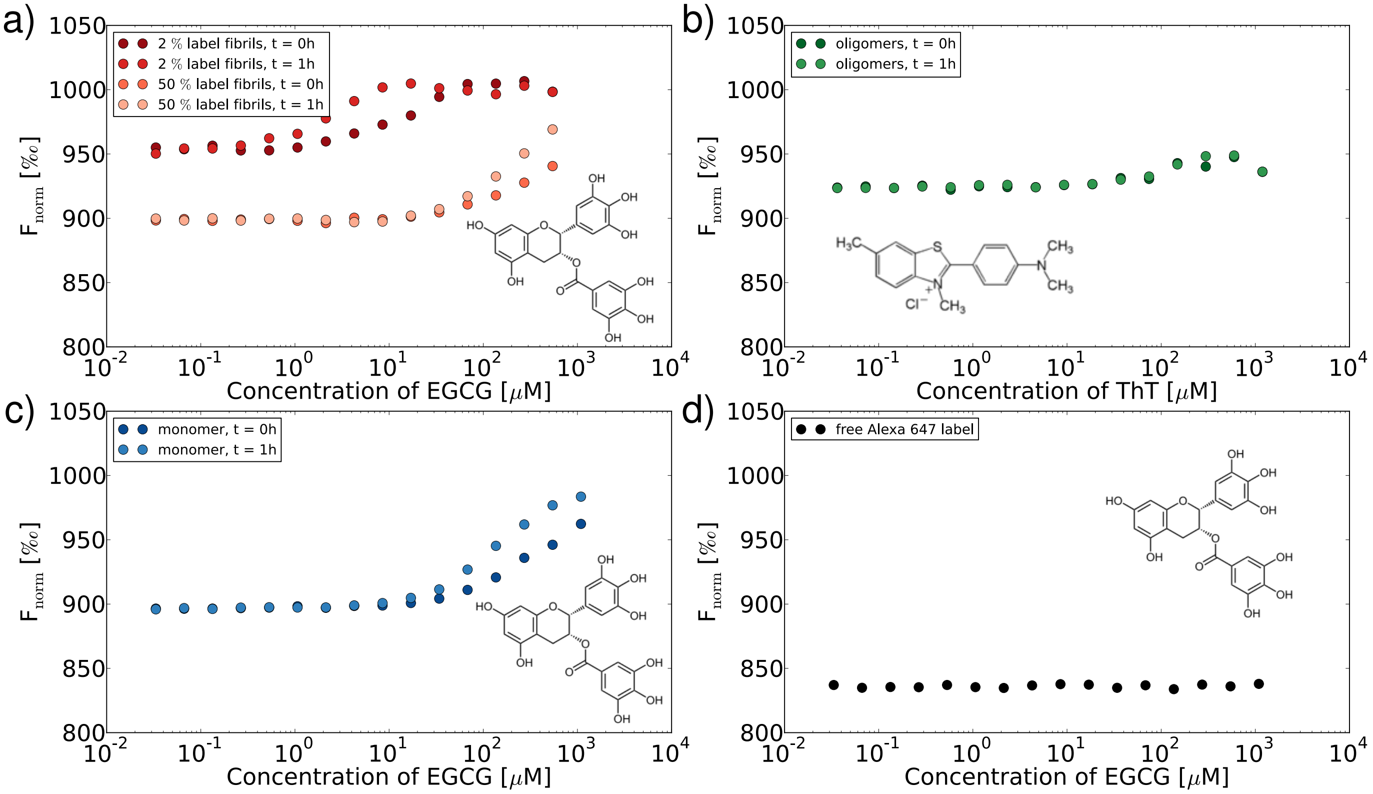

Figure 6: a) α-synuclein amyloid fibrils with either 2% or with 50% of the constituent monomers labeled with Alexa 647 are incubated with increasing concentrations of EGCG. Thermophoresis measurements were performed immediately after sample preparation and again after 1 h incubation period. It is apparent from this data that the labeling density is an important experimental parameter, and that it should be minimized as much as possible. b) α-synuclein oligomers show a weak interaction with Thioflavin-T, that does not change over time. c) Monomeric α-synuclein shows a weak interaction with EGCG, which also displays some time dependence. d) Free Alexa Fluor® 647 malimide does not display any interactions with EGCG.

5.1 Data analysis

We follow here the procedures outlines in [13]. The normalized correlation function G(τ) is defined as:

$$\frac{\langle F(t)F(t+\tau)\rangle}{\langle F(t)\rangle^2} \tag{1}$$

where angular brackets denote the average over time, F(t) the fluorescence signal at time t and F(t+τ) the fluorescence signal at a later time t+τ. An ideal three dimensional Gaussian shape is assumed for the confocal volume. The structure parameter ω describes the ratio of the half axis z_0 to the radius of the laser beam w_0. By fitting a model to the experimental data, physically relevant information can be extracted from the correlation curve. The autocorrelation curve for a single component freely diffusing in a 3D Gaussian element can be described by:

$$G(\tau) = \frac{1}{N}\left(\frac{1}{1+\frac{\tau}{\tau_D}}\right)\left(\frac{1}{1+\frac{\tau}{\omega^2\tau_D}}\right)^{\frac{1}{2}} + 1 \tag{2}$$

where N is the number of particles inside the confocal volume, τ_D is the translational diffusion time of the species, τ is the correlation time and ω the structure parameter. For samples containing two components of different size a two component fit is used:

$$G(\tau) = \frac{1}{N}\left[(1-y)\left(\frac{1}{1+\frac{\tau}{\tau_{D1}}}\right)\left(\frac{1}{1+\frac{\tau}{\omega^2\tau_{D1}}}\right)^{\frac{1}{2}} + y\left(\frac{1}{1+\frac{\tau}{\tau_{D2}}}\right)\left(\frac{1}{1+\frac{\tau}{\omega^2\tau_{D2}}}\right)^{\frac{1}{2}}\right] + 1 \tag{3}$$

where τ_{D1} and τ_{D2} are the diffusion times of the two components and y is the fraction of the second component. To take optical dark states of the dye into account, a function that describes the triplet can be integrated into the fitting equation:

$$G_{\text{Triplet}}(\tau) = \left(1 + \frac{T}{1-T}\exp\left(-\frac{\tau}{\tau_T}\right)\right) \tag{4}$$

τ_T is the triplet state relaxation time and T the fraction of fluorophores in the dark state. The total correlation curve then becomes a product of the triplet function and the model G (τ):

$$G_{\text{total}}(\tau) = G_{\text{Triplet}}(\tau)G(\tau) \tag{5}$$

The translational diffusion time describes the average dwell time of a molecule with diffusion constant D in the confocal volume:

$$\tau_D = \frac{{w_0}^2}{4D} \tag{6}$$

The hydrodynamic radius R_h of a spherical molecule can be determined with the Stokes-Einstein-equation:

$$R_h = \frac{k_B T}{6\pi\eta D} \tag{7}$$

where k_B, is the Boltzman constant, T is the temperature in [K] and η the viscosity of the surrounding medium.

5.2 Results

We have determined the sizes of fluorescently labeled monomeric, oligomeric and fibrillar α-synuclein using FCS. Figure 7 shows the normalized correlation functions that clearly illustrate the difference in diffusion behavior between the different species.

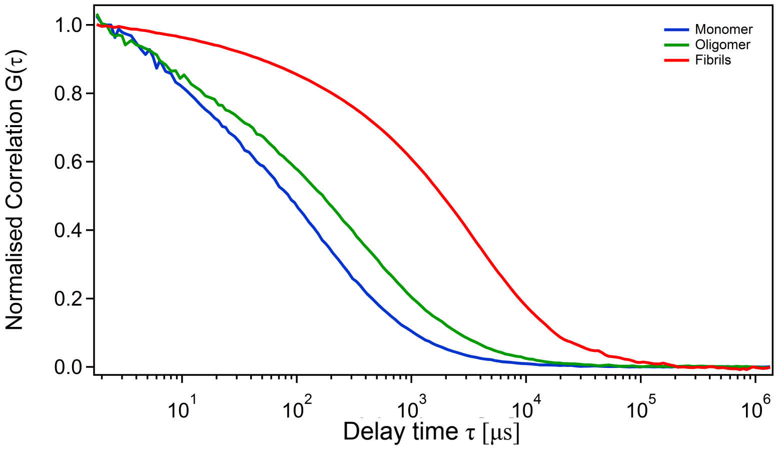

Figure 7: Normalized correlation curves of monomeric (blue), oligomeric (green) and fibrillar (red) α-synuclein. FCS measurements enable accurate determination of the diffusion coefficient and hence the (effective) hydrodynamic radii of the different species, using the Stokes-Einstein equation (Equation 7). The shift to the right towards longer diffusion times is an indicator for an increase in size.

The experiment shown in Figure 7 yields values of the diffusion coefficient of $8.2\pm0.34\cdot10^{-11}\ \frac{m^2}{s}$ (monomer), $3.5\pm0.34\cdot10^{-11}\ \frac{m^2}{s}$ (oligomer) and $4.1\pm0.4\cdot10^{-12}\frac{m^2}{s}$ (fibrils). In the case of the monomers and oligomers, the Stokes-Einstein equation (Eq. 7) can be directly applied in order to determine the hydrodynamic radii, as these species can be approximated as spheres. As averages from several independent experiments, we obtain $R_M = 2.8\pm0.1$ nm for the hydrodynamic radius of the monomer and $R_O = 7.5\pm0.7$ nm for the hydrodynamic radii of the oligomers. The average diffusion coefficient measured for the sonicated fibrils is $4.1\pm0.4\cdot10^{-12}\ \frac{m^2}{s}$. In all cases when fibrillar samples were measured by FCS, the quality of a two-component fit was considerably better, where the second component was fixed to have the size of the monomer. The monomer component of the fibril samples varied between 15 and 23%, which also introduces an error into the values of the Soret coefficients measured for the fibrils. In order to calculate the diffusion coefficient

and subsequently the dimensions of the fibrils, we make use of the AFM images that we have taken of these fibrils (see Figure 3 of main manuscript). These images in principle already provide us with the necessary information about the dimensions of the fibrils, but we will nevertheless check whether the results of the FCS measurements that are performed under the same conditions as the thermophoresis and electrophoresis experiments, are consistent with the AFM images. According to the detailed analysis of the AFM images, the sonicated fibrils have an average length of 171 nm and a thickness of approximately 8 nm. With these dimensions, we can calculate the rotational diffusion coefficient, according to:

$$D_r = \frac{k_B T}{\gamma_r} \tag{8}$$

where γ_r is the rotational friction coefficient, given according to Broersma [14] by:

$$\gamma_r = \frac{\pi \eta L^3}{3(\log(2L/D) - c_r)} \tag{9}$$

where L is the length of the rod, D is its diameter and c_r is a numerical factor that corrects for end effects:

$$c_r = 1.57 - 7\left(\frac{1}{\log(2L/D)} - 0.28\right)^2 \tag{10}$$

The rotational diffusion coefficient has units of radians^2/s. We obtain a value of $\sim$ 1725 rad^2/s. The diffusion time of a fibril through the confocal volume of the FCS instrument is approximately 5 ms, a value directly obtainable from the FCS measurements. During this time, the fibril will rotate on average about 168°. This is probably not enough to assume complete orientational averaging, but some degree of averaging can be assumed. We can therefore expect the difference between the measured apparent diffusion coefficient of the fibrils and the calculated diffusion coefficient of a rod with 171 nm length and 8 nm thickness to be not too large. In order to calculate the translational diffusion coefficient of a rod, we use the results by de la Torre [15]:

$$D_t = \frac{k_B T(\log(L/D) + c_t)}{3\pi\eta L} \tag{11}$$

where c_t is again a numerical correction factor for end effects:

$$c_t = \frac{c_{t,\perp} + c_{t,\parallel}}{2} \tag{12}$$

with

$$c_{t,\perp} = 0.5 + 4.2\left(\frac{1}{\log(2L/D)} - 0.39\right)^2 \tag{13}$$

and

$$c_{t,\parallel} = -0.58 + 7.4\left(\frac{1}{\log(2L/D)} - 0.34\right)^2 \tag{14}$$

We obtain $c_t = 0.0122$ and $D_t = 8.1 \cdot 10^{-12}\ \frac{m^2}{s}$. This value is in acceptable agreement with the value determined from FCS ($4.1 \pm 0.4 \cdot 10^{-12}\ \frac{m^2}{s}$, see above). The difference in these values is likely to stem from the polydispersity of the fibrillar sample. The FCS measurements are likely to be biased towards the shorter fibrils, due to faster diffusion and less likely absorption on the walls etc., whereas the AFM measurements might be biased towards the longer fibrils, due to preferential absorption on the AFM substrate.

We have also performed FCS measurements of α-synuclein fibrils before and after incubation with additional unlabeled monomeric protein at 70 °C, as in the experiment shown in Figure 3 of the main manuscript. The correlation data of this experiment is shown in Figure 8, and the growth of the fibrils is clearly apparent from this data.
Finally, we also used FCS in order to probe the temporal stability of the purified oligomers. We measured a sample of oligomers continuously for more than 60 h at RT (Figure 9) and found only a very slight trend towards increasing sizes. This result confirms that the oligomers are thermodynamically highly stable and display negligible kinetics of dissociation on the time scale of days. Therefore purified samples of oligomers were stored during up to several days (at RT or 4 °C) and used for thermophoresis experiment throughout this period.

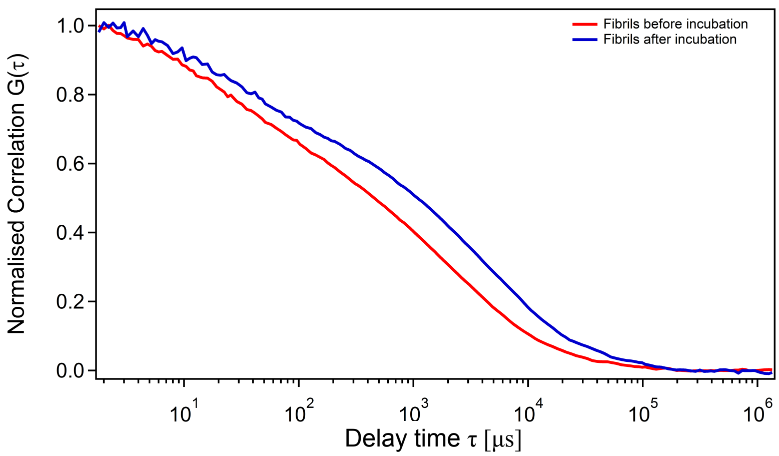

Figure 8: Normalized correlation curves from FCS experiments of α-synuclein amyloid fibrils before and after a 20 min incubation period with 70 μM added unlabeled monomeric protein at 70 °C. The shift to the right towards longer diffusion times is an indicator for an increase in size. This shift corresponds to a change in diffusion coefficient from $3.8 \pm 0.2 \cdot 10^{-12}\ \frac{m^2}{s}$ to $2.4 \pm 0.2 \cdot 10^{-12}\ \frac{m^2}{s}$.

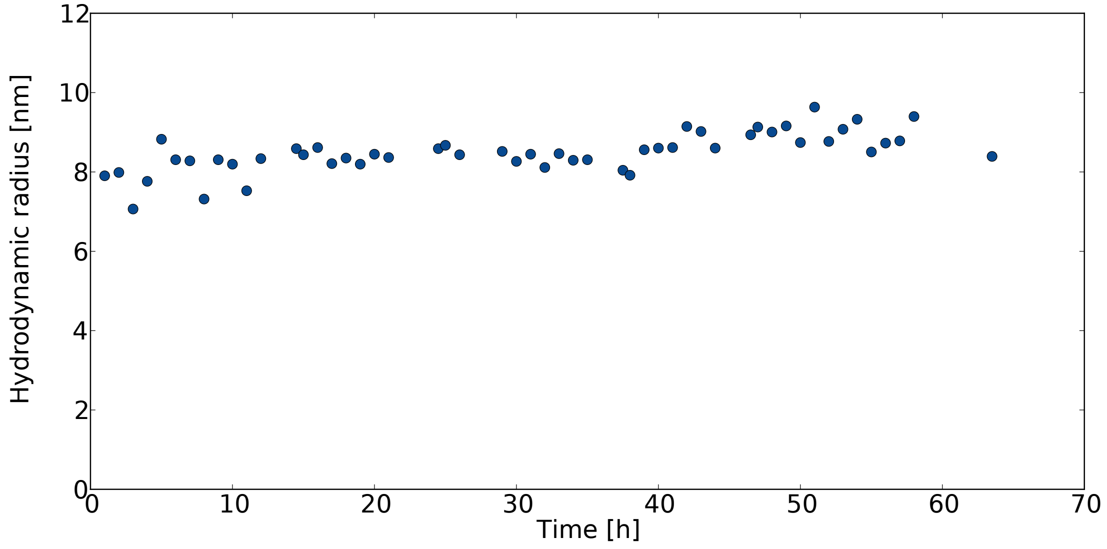

Figure 9: Time course of FCS size measurements of labeled α-synuclein oligomers over more than 60 h, confirming the high structural stability of these types of aggregates.

6 Free flow electrophoresis measurements of monomeric and aggregated α-synuclein

As will be discussed in detail in the next section, the net charge and electrophoretic mobility are important parameters for the quantitative description of thermophoresis. The thermophoresis of the salt ions in solution creates a macroscopic electric field at steady state that leads to electrophoresis of the macromolecule under study, such as the monomeric or aggregated α-synuclein in the present case. Furthermore, the potential energy stored in the double layer depends on both the charge and the temperature and hence yields another temperature-dependent contribution to the Soret coefficient. Therefore, independent estimates of the effective charges of the different α-synuclein species are important for the quantitative analysis and understanding of the thermophoretic behavior.

We used a variant of microfluidic free flow electrophoresis that was recently developed in our laboratory [16] in order to measure the electrophoretic mobilities of monomeric, oligomeric and fibrillar α-synuclein in 5 mM Tris buffer at pH 7.4 (Figure 10).

The measured electrophoretic mobilities are -2.06±0.11 $\frac{\mu m/s}{V/cm}$ (labeled monomers), -3.09±0.46 $\frac{\mu m/s}{V/cm}$ (oligomers) and

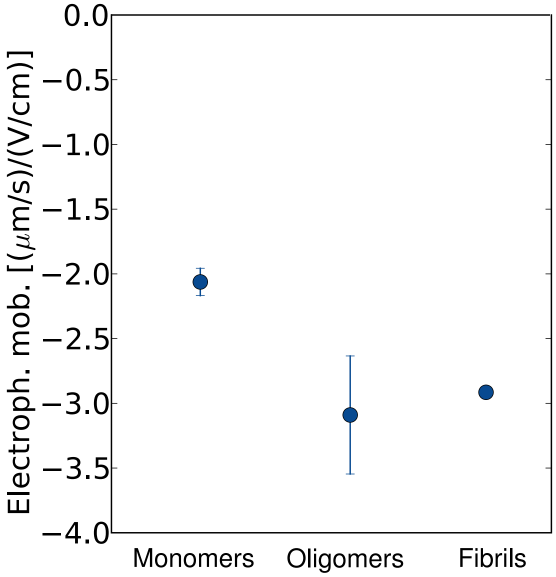

Figure 10: Electrophoretic mobilities, measured via microfluidic free-flow electrophoresis [16]. The mobilities were measured in 5 mM Tris buffer, pH 7.4. Error bars come from two independent repeats with independent sample preparations.

-2.91±0.02 $\frac{\mu\text{m/s}}{\text{V/cm}}$ (fibrils). The values for both monomeric and fibrillar α-synuclein are well-defined and display a trend of increasing mobility with increasing size. However, the variation in the values measured for the oligomers is much larger, potentially reflecting a degree of polydispersity of the oligomer preparations [4]. In particular, oligomeric and fibrillar α-synuclein display an essentially identical electrophoretic mobility. In the light of the very different sizes and structures of oligomers and fibrils, this is a very interesting finding which highlights the power of thermophoresis to discriminate more easily between different types of particles, compared to electrophoresis. For our quantitative modeling of the thermophoresis data, we require the surface charge density or the absolute charge of the protein monomers and aggregates, in addition to the electrophoretic mobilities. A comprehensive theoretical framework for relating mobilities to surface potentials for colloid-like systems of all sizes has been first presented by Henry [17]. In particular for particles that can be approximated as spheres, such as monomeric and oligomeric α-synuclein, this is well-established.

6.1 From electrophoretic mobility to effective charge for monomeric and oligomeric α-synuclein

The ζ-potential is the potential difference between the (neutral) bulk solution and the surface of a charged particle, including tightly absorbed counter ions [18], and is therefore a measure for the charge density of the particle surface. The electrophoretic mobility and the ζ-potential are related through Henry's formula [17]:

$$\mu_e = \frac{2\epsilon_0\epsilon_r\zeta f(\kappa R)}{3\eta} \tag{15}$$

where ζ is the ζ-potential, η the viscosity and $f(\kappa R)$ is Henry's function with the argument κR, the product of inverse Debye length κ and particle radius R. Henry's function varies from $1.0 \leq f(\kappa R) \leq 1.5$. For $\kappa R \ll 1$, $f(\kappa R)$ =1.0 (Hückel limit) and for $\kappa R \gg 1$, $f(\kappa R) = 1.5$ (Smoluchowski limit). Ohshima has presented a formula for f(κR) that is valid for all values of κR and accurate to within 1% [19]:

$$f(\kappa R) = 1 + 0.5\frac{1}{(1 + \frac{2.5}{\kappa R(1+2e^{-\kappa R})})^3} \tag{16}$$

However, the applicability of Henry's expression for the particle mobility depends on the absolute value of the charge and mobility, due to the fact that Henry's treatment neglects relaxation effects of the counterion atmosphere [18]. We can define a reduced mobility:

$$\mu_{\text{red}} = \frac{3\eta e}{2\epsilon_0\epsilon_r k_B T}\mu_e \tag{17}$$

Depending on the relative values of μ_{red} and κR, either Henry's simplified treatment or a more sophisticated one has to be applied. We obtain $\mu_{\text{red,M}}$ = -1.74 and $\mu_{\text{red,O}}$ = -2.62. From the FCS measurements (see above), we know that the radius of the monomeric protein, R_m, is ca. 2.8 nm and the radius of the oligomers, R_o, is ca. 7.5 nm. The free flow electrophoresis experiments were carried out in 5 mM Tris buffer pH 7.4, where the Debye length $\lambda = 1/\kappa$ = 4.7 nm. Hence κR = 0.6 for monomeric α-synuclein and 1.6 for oligomeric α-synuclein. It turns out that Henry's simplified treatment for the calculation of the ζ-potential is applicable for the monomer (with f(κR)=1.02), but for the oligomer, we need to use Hunter's inversion [18] of Booth's formula [20]:

$$\zeta_r = \frac{\mu_{\text{red}}}{f(\kappa R)} - \left[\frac{C_3(\mu_{\text{red}}/f(\kappa R))^3 + C_4(\mu_{\text{red}}/f(\kappa R))^4}{f(\kappa R) + 3C_3(\mu_{\text{red}}/f(\kappa R))^2 + 4C_4(\mu_{\text{red}}/f(\kappa R))^3} \right] \tag{18}$$

where ζ_r is the reduced ζ-potential:

$$\zeta_r = \frac{\zeta e}{k_B T} \tag{19}$$

and where C_3 and C_4 are numerical coefficients that depend on the product κR and the limiting molar conductance of the counter- and coions. Hunter [18] gives the expressions for these coefficients that we are not reproducing here. The limiting conductances of the $Tris^+$ ion and the Cl^- ion are $2.97\cdot10^{-3}\Omega^{-1}m^2mol^{-1}$ [21] and $7.64\cdot10^{-3}\Omega^{-1}m^2mol^{-1}$ [22].

We are now in a position to calculate the ζ-potentials for monomeric (44.0 mV) and oligomeric (40.3 mV) α-synuclein. In order to calculate the charges from these values of the ζ-potentials, we follow the methodology outlined by Ohshima [23]. We define the dimensionless surface charge density Σ:

$$\Sigma = \frac{e\sigma}{\epsilon_0 \epsilon_r \kappa k_B T} \tag{20}$$

where σ is the surface charge density. Ohshima derives the following expression relating reduced ζ-potential and reduced surface charge density:

$$\Sigma = 2\sinh(\zeta_r/2) \left[1 + \frac{2}{\kappa R \cosh^2(\frac{\zeta_r}{4})} + \frac{8\log(\cosh(\frac{\zeta_r}{4}))}{(\kappa R)^2 \sinh^2(\frac{\zeta_r}{2})} \right]^{\frac{1}{2}} \tag{21}$$

The absolute charge Q can be computed from the surface charge density σ as Q = $4\pi R^2\sigma$. Using these expressions, we obtain values for the absolute charge of Q_M = -10.9 e for the monomeric protein and Q_O = -50.4 e for the oligomers. The value of the monomer is in good agreement with theoretical predictions, based on the combined charge of the individual amino acids at this pH value (-9.1 e) and the charge of the Alexa 647 label (-4 e). For the oligomers, on the other hand, the effective charge corresponds to only ~5 times the charge of a monomer, while each oligomer consists on average of ~30 monomers. This strong difference can be explained through processes such as counter ion incorporation into the oligomer structure, as well as Manning condensation of counterions [24] onto the surface of the oligomer. Indeed, without such charge compensation mechanisms, the electrostatic contribution to the formation of an oligomer would render such a structure highly energetically unfavorable.

6.2 Free flow electrophoresis of rod-like particles

The determination of effective charges from electrophoretic mobilities of rod-like particles represents an additional difficulty, due to the anisotropic nature of the rod and the possibility of alignment in the electric field. In addition, if the electrophoretic mobility is measured in free (shear) flow, alignment could also occur in the flow direction. We start by evaluating whether or not alignment effects have to be taken into account in our setup. Dhont and Briels [25] have given a comprehensive treatment of the behavior of Brownian rods in shear flow. A rotational Péclet number can be defined that quantifies the relative importance of shear forces and random Brownian rotational motion:

$$\text{Pe}_r = \frac{\dot{\gamma}}{D_r} \tag{22}$$

where $\dot{\gamma} = \frac{du_x}{dy}$ is the shear rate, u_x is the flow velocity and y is a direction perpendicular to the flow. The microfluidic channel is 2200 µm wide and 25 µm high. It is therefore clear that in the horizontal direction, the shear rate will be negligibly small in the center of the channel, while it might be considerable in the vertical direction. Therefore we limit our treatment to this direction. The flow rate is 500 µl per hour, corresponding to an average flow velocity of $2.5\cdot10^{-3}\frac{m}{s}$. We take the flow rate to be approximately constant throughout the wide dimension of the channel and approximate the parabolic flow profile across the narrow dimension by a linear one, and therefore the maximum flow velocity is twice the average flow velocity. Hence we obtain a shear rate of $4\cdot10^2 s^{-1}$. The rotational diffusion coefficient

has been determined above to be 1700 $\frac{\text{rad}^2}{s}$, and hence we obtain $\text{Pe}_r \approx 0.24$. This is smaller than the threshold for the beginning of orientation, $\text{Pe}_r \sim 1$ [26]. Therefore, shear alignment is negligible in our setup for short amyloid fibrils.

Next, we investigate whether the α-synuclein fibrils can be aligned by the electric field applied during the electrophoretic measurements. Alignment in an electric field can occur by means of a permanent or an induced electric dipole. We can apply phenomenological, as well as theoretical arguments. Phenomenologically, the electrophoretic mobility is expected to increase with the applied voltage in the case of significant alignment, as the degree of alignment should increase with the field strength and aligned rods have a lower friction factor - this argument has been given in favor of alignment in the case of tobacco mosaic viruses [27]. However, as can be seen in Figure 11, the electrophoretic velocity increases linearly with the electric field, and therefore an increase in mobility is not detected. This observation hints towards no significant alignment effects.

Next, we treat the question of whether alignment by the field plays a role or not theoretically. The first question is whether the fibrils possess a permanent dipole moment. Measurements of permanent dipole moments of rod-like protein aggregates are rare. It has been reported that β-lactoglobulin amyloid fibrils with an average length of ca. 4 µm have a permanent dipole moment of $3.1 \cdot 10^{-19}$Cm. This value, which agrees remarkably well with the value calculated for a chain-like assembly of monomers, has been obtained from electric field-induced birefringence at pH 2.0, where the protein carries a significant net charge of $\sim$+20 e. On the other hand, it has been reported that the tobacco mosaic virus, a rod-like structure of $\sim$300 nm in length and 18 nm in diameter, possesses a permanent dipole moment of only $8.3 \cdot 10^{-26}$Cm [28] at pH 7.5, where the virus coat protein carries a net charge of -3.4 e. It therefore seems likely that the α-synuclein fibrils possess a permanent dipole moment, but it is difficult to estimate its magnitude, especially as no detailed structural information is available for the fibrils.

Furthermore, fibrillar protein structures display a polarizability due to the mobile counterions that are associated with the charged rod, leading to an induced dipole moment. An expression for this induced dipole moment has been given by Fixman [29, 30]:

$$\mu_{\text{ind}} = \frac{4\pi\epsilon_0\epsilon_r LKz_1E_0}{\gamma^2(z_1 - z_2)}\left(1 - \frac{\tanh(\gamma L/2)}{\gamma L/2}\right) \tag{23}$$

where z_1 and z_2 are the counter- and co-ion valencies and γ is a characteristic length scale:

$$\gamma^2 = \frac{4\pi c_1 Kb}{\phi} \tag{24}$$

where c_1 is the bulk concentration of counter ions, K is a numerical factor, given by:

$$K = \frac{1}{2\log(2L/D) - 14/3} \tag{25}$$

The fraction of bound counter ions ϕ depends on the mean spacing between charges on the rod-like macromolecule, b, and the Bjerrum length, $\lambda_B = \frac{e^2}{4\pi\epsilon\epsilon_0 k_B T}$, according to:

$$\phi = 1 - \frac{b}{\lambda_B} \tag{26}$$

With a fibril diameter of 8 nm, a fibril density of 1.35 g/cm^3, a molecular mass and charge of the α-synuclein monomer of 14.46 kDa and -9 e, respectively, we obtain a mean spacing of the charges of 0.4 Å. The Bjerrum length in water at room temperature is $\sim$0.7 nm and hence $\phi \approx 0.945$. Using those values, we obtain an induced dipole moment of $\sim 1.1 \cdot 10^{-27}$ Cm. This value is negligible compared to the potential permanent dipole moments mentioned above. We can now compute the orientational order parameter Φ ($\Phi \in [0,1]$) for various values of μ_0, the permanent dipole moment. The orientational order parameter can be calculated as [31]:

$$\Phi = 1 - 3\frac{\coth(\beta) - 1/\beta}{\beta} \tag{27}$$

with β defined as $\beta = \frac{\mu_0 E}{k_B T}$. If we take the range of values for μ_0 from the ones reported above: 10^{-25} Cm$< \mu_0 < 10^{-20}$ Cm, we obtain values of Φ that vary between 0.0008 and 0.9997, and hence between negligible and complete alignment. Therefore it is impossible to say from theoretical arguments alone, without a more precise estimate of the fibrils' permanent dipole moment, whether or not alignment with the field direction occurs. We will therefore analyze the electrophoresis data assuming complete and no alignment and compare the results. Expressions relating the electrophoretic mobilities of aligned and randomly oriented rods have been given by Ohshima [32]:

$$\mu_{\|} = \frac{\epsilon_0\epsilon_r}{\eta}\zeta \tag{28}$$

and

$$\mu_{\text{rand}} = \frac{\epsilon_0 \epsilon_r}{3\eta} \zeta(1 + 2f(\kappa R)) \tag{29}$$

where f(κR) is Henry's function for a cylinder, that varies between 0.5 and 1. Therefore

$$\frac{\mu_{\|}}{\mu_{\text{rand}}} = \frac{3}{1 + 2f(\kappa R)} \tag{30}$$

varies between 1.5 and 1.0. Henry's function for a cylinder is given by [32]:

$$f(\kappa R) = 0.5 + 0.5 \frac{1}{(1 + \frac{2.55}{\kappa R(1+e^{-\kappa R})})^2} \tag{31}$$

which yields f(κR) = 0.55 in the case of fibrils of a radius of R = 4 nm in 5 mM Tris buffer. With this value of f(κR), we can calculate the ζ-potential for the cases of complete alignment (42.2 mV) and random orientation (60.2 mV). Hunter [18] gives the relationship between the total charge of a cylinder and the ζ-potential:

$$\zeta = \frac{2 Q_F K_0(\kappa R)}{4\pi \epsilon_0 \epsilon_r \kappa R (2R + L) K_1(\kappa R)} \tag{32}$$

where $K_0(\kappa R)$ and $K_1(\kappa R)$ are the modified Bessel functions of the second kind ($K_0(\kappa R)/K_1(\kappa R) \approx 0.85$ under these conditions [18]). Using this relationship, we can calculate average total charges of the fibrils of -205 e (aligned case) and -292 e (random orientation). Both of these values are in good agreement with a rough estimate of the total charge based on the fraction of condensed counter ions calculated above. A fraction of bound counterions of 0.945 leaves the fibrils with a net charge of -238 e. This analysis is therefore consistent with both aligned and randomly oriented fibrils and we conclude that the fibrils possess a net charge of between 200-300 e.

6.3 Electrophoresis under different solution conditions

In order to check this analysis for its robustness and consistency, we have also performed similar electrophoresis experiments in 10 mM Tris buffer instead of 5 mM Tris. We obtain electrophoretic mobilities of -1.20 $\frac{\mu\text{m/s}}{\text{V/cm}}$ and -1.95 $\frac{\mu\text{m/s}}{\text{V/cm}}$ for monomeric and oligomeric α-synuclein under these conditions. This translates into charges of -7.2 e and -37.3 e. This apparent decrease in charge compared to the conditions of lower ionic strength might have its origin in more pronounced counter-ion binding, but could also stem from the fact that the measurement become more difficult and inaccurate at higher ionic strengths. The contribution of the fluorescent sample to the overall current, and hence the measured deflection of the sample beam, decreases with increasing solution ionic strength.

Furthermore, we have also performed experiments in 5 mM Tris buffer in the presence of 100 µM EGCG, and we found that the measured values for the mobilities are almost indistinguishable in the presence and absence of EGCG for all α-synuclein species.

7 Modeling electrostatic effects in protein thermophoresis

One of the aims of this study is to be able to quantitatively understand and model the effects of changes in solution conditions on the thermophoresis of proteins. We have recently presented an analysis of nucleic acid thermophoresis that highlighted the importance of electrostatic effects [11]. We apply this model to the thermophoresis of proteins and protein aggregates in the present study.

In our model the Soret coefficient of a biopolymer consists of three main contributions. For charged biomacromolecules, the capacitor effect $\left(S_T^{CM}\right)$ and Seebeck effect $\left(S_T^{EL}\right)$ play an important role.

$$S_T = S_T^{CM} + S_T^{EL} + S_T^{NI} \tag{33}$$

The nonionic contribution $\left(S_T^{NI}\right)$ cannot, at present, be modeled and is likely to involve surface properties of the molecule undergoing thermophoresis, such as its hydophobicity. We shown in the present study that changes in such properties lead to changes in thermophoretic behavior (e.g. the binding of the neutral molecule EGCG to α-synuclein oligomers and fibrils).

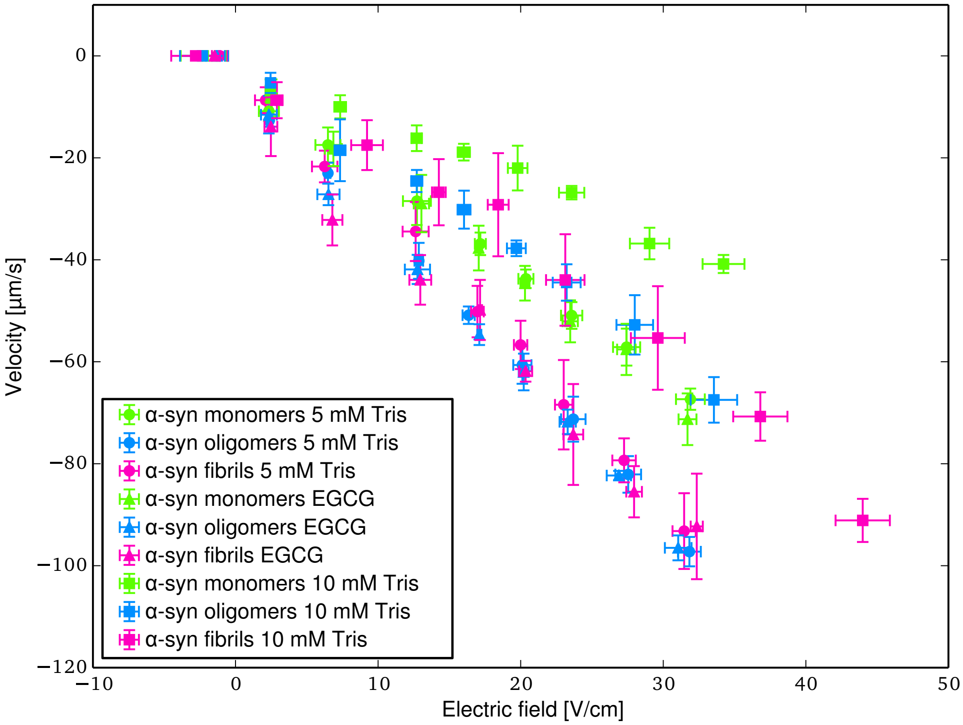

Figure 11: Plot of electrophoretic velocities vs. electric field. Shown is data for monomeric, oligomeric and fibrillar α-synuclein, in 5 and 10 mM Tris buffer, pH 7.4, as well as in 5 mM Tris buffer in the presence of 100 µM EGCG.

7.1 Capacitor effect

One important contribution to S_T of charged macromolecules is given by the capacitor effect S_T^{CM}. It results from the change in free energy of the molecule's ion cloud upon temperature variation and is dependent on the properties of the Debye layer [11, 33]. The crucial parameters are the Debye length λ_D, as well as the polymer's charge Q and radius R. In addition the density ρ, permittivity $\epsilon{=}\epsilon_0\epsilon_r$ and temperature T of the solvent water affect the magnitude of S_T^{CM}. Therefore the Soret coefficient of a charged biopolymer at any given temperature is a function of the ionic strength. For spherical particles or molecules, as monomers and oligomers the capacitor model yields a contribution to S_T of:

$$S_T^{CM} = \frac{(Qe)^2}{16\pi k_B T^2 \epsilon \lambda_D \left(1 + R/\lambda_D\right)^2} \left(1 - \frac{\partial \log[\rho(T)]}{\partial \log[T]} - \frac{\partial \log[\epsilon(T)]}{\partial \log[T]} \left(1 - \frac{2\lambda_D}{R}\right)\right) \tag{34}$$

For rodlike structures, such as amyloid fibrils, the capacitance can be expressed as a superposition of a spherical and a cylindrical capacitor [34].

7.2 Seebeck effect

The salt ions in solution have themselves different Soret coefficients and are therefore affected differently by thermophoresis. As a consequence, a macroscopic electric field builds up if a thermal gradient is applied to a salt solution [11, 35, 36]. The charged macromolecules under study will undergo electrophoresis in the electric field thus created. This dependence, which in analogy to solid state thermoelectric phenomena has been dubbed the Seebeck effect, can be expressed as:

$$S_T^{EL} = -\frac{k_B T \mu}{eD} \frac{\sum_i z_i c_i S_{Ti}}{\sum_i z_i^2 c_i} \tag{35}$$

The mobility is related to the zeta potential via Eq. 15 and $\zeta = \frac{Qe}{4\pi\varepsilon r(1+r/\lambda)}$. For the Soret coefficients of single ions in water, S_{Ti}, literature values according to the reduction rule were applied ([37] and see also Reichl et al. [11])

$$S_T^{Cl^-} = 7.18 \cdot 10^{-4}/K \tag{36}$$

$$S_T^{Na^+} = 4.69 \cdot 10^{-3}/K \tag{37}$$

7.3 Nonionic contribution

The non-ionic contribution to the Soret coefficient of biomacromolecules is likely to be linked to surface properties of the molecule, such as hydrophobicity and hydrogen bonding capacity with water [10]. At present, no general framework exists that is able to describe these effects quantitatively. Therefore we restrict our theoretical description to the ideal gas contribution and an empirical temperature dependent part according to Piazza [38].

$$S_T^{NI} = \frac{1}{T} + S_T^{\infty}(1 - e^{-(T-T_0)/T^*}) \tag{38}$$

Here S_T^{∞} denotes the empirical Soret coefficient at infinite temperature, T_0 the temperature where the Soret coefficient changes sign and T^* is a measure for the broadness of the range where S_T increases with temperature.

7.4 Determination of 'thermophoretic charge' and nonionic contribution

On the basis of the model for the electrostatic contributions to the Soret coefficient of the protein molecules and aggregates outlined above, the 'thermophoretic charges', Q_T, and nonionic contributions, S_T^{NI}, are determined from the ionic strength series for the monomeric and oligomeric α-synuclein species (Figure 2 of the main manuscript). Taking into account the temperature dependent physical parameters of water (viscosity, permittivity, density), as well as salt concentration, concentration and size of the protein species an optimization of χ^2 for the measured data yields the parameters listed below for monomers and oligomers. Unfortunately it was not possible to perform measurements of the Soret coefficient as a function of the ionic strength for the α-synuclein amyloid fibrils due to higher order assembly of the fibrils at higher ionic strengths [5] and sticking interactions with the capillary walls.
For Soret coefficient of the Tris ion, individual parametric sweeps were performed on the salt series of monomeric and oligomeric α-synuclein. For the two α-synuclein species the best fits yields $S_T(Tris)^{Mon}$=0.0024/K and $S_T(Tris)^{Oligo}$=0.0038/K. Consequently the Soret coefficient was set to the median of both measurements $\overline{S_T(Tris)}$=0.0031/K. This value is reasonable since it is quantitatively comparable to the one of sodium with 0.00469/K [11, 37].

The best fit of the model to the data yields the following effective thermophoretic charges and nonionic contributions for monomeric and oligomeric α-synuclein:

	Q_T	$S_T^{\infty}[1/K]$	$T_0[K]$	$T^*[K]$
Monomer	-6.9 e	0.0126	292.4	20.7
Oligomer	-29.2 e	0.0161	320.6	33.4

Nonionic Contribution: Interestingly S_T^{NI} is higher for the monomers in the observed temperature range, but increases more strongly for the oligomers over base temperature (Figure 12). Whether hydrophobic effects play a role here is not clear. It is also under discussion whether the strong temperature dependence of the empirical nonionic contribution is connected to additional electrostatic effects not currently included. The thermophoresis of salt ions changes over temperature [39] and can result in further diffusiophoretic or electrophoretic influence on S_T of the macromolecule [36].
Thermophoretic charge Q: Overall the obtained charges on the basis of thermophoresis measurements are significantly smaller than the charges determined from electrophoretic measurements: Q_T^{Mon} =-6.9 e, Q_T^{Oligo} =-29.2 e from thermophoresis vs. Q_E^{Mon} =-10.9 e, Q_E^{Oligo} =-50.4 e from electrophoresis. This difference might originate in an incomplete description of the electrostatic effects involved in thermophoresis and does indeed provide an important comparison that will be helpful for further theoretical studies of protein electrophoresis and thermophoresis.
Influence of unlabeled monomers (Fig 3a of main manuscript): The Soret coefficient of the additional unlabeled monomer was set according to the expected contribution for labeled monomers in 1mM Tris: $S_T^{unl.mon} = \overline{S_T^{NI-mon.} + S_T^{CM-mon.}} = 0.031/K$. Note that this Soret coefficient corresponds to the median value over the observed temperature range in Figure 3a of main manuscript. The monomers were then incorporated as additional salt species in the Seebeck effect and taken into account for the the Debye length (Capacitor Model). The observed decrease in the Soret coefficient of the labeled monomers and oligomers in the presence of unlabeled monomers coincides with similar findings on DNA [34] in the dilute regime.
Influence of the nanobody Syn2 (Fig 4a of main manuscript): In the case of nanobodies present, the best fit to the data yields the following charges:

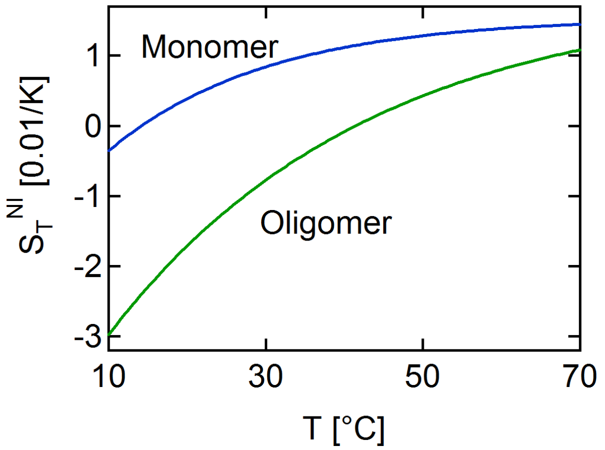

Figure 12: The non-ionic contribution to the Soret coefficient, S_T^{NI}, for monomeric and oligomer α-synuclein as a function of temperature.

	Monomer	Oligomer
No nanobody	-4.5 e	-27.6 e
Nanobody	-1.8 e	-20.6 e

The reason for the differences in charge listed here in the absence of nanobody and the charge determined for the monomers and oligomers stated above is likely to be due to the modified solution conditions (presence of phosphate ions in the nanobody solution and respectively also added to the control) and the associated altered Seebeck effect. For monomers, we obtain a $\Delta Q = +2.7$ e, which is in reasonable agreement with the expected binding of one nanobody (+1.5 e). In the case of the oligomers, the observed value of $\Delta Q = +7$ e is consistent with the attachment of more than one molecule of nanobody. It is difficult to estimate the expected stoichiometry of binding of nanobody to the oligomers, due to lack of detailed structural information of the oligomer and hence of the accessibilities of the epitopes.

8 ComSol simulations of the thermophoresis setup

The Soret coefficient determined from the experimentally measured concentration depletion value depends linearly on the magnitude of the induced temperature jump ΔT. In order to obtain insight into the experimental uncertainty of the induced temperature gradient, we performed a finite element simulation and compared the results with the measured values for ΔT. The decrease of ΔT with increasing base temperature T for simulation is in good agreement with the measurements. Furthermore the effect of errors in capillary thickness (±10%) could be tested in the simulations and the resulting error for ΔT deduced.
The simulation was performed in COMSOL Multiphysics (COMSOL Inc., USA) whereby the physics of infrared-absorption and heat transfer were incorporated (Figure 13 a). In our simulation we assumed radial symmetry and incorporated the capillary with dimensions of 50 µm x 500 µm. The capillary is coupled to a silicon wafer of 350 µm thickness with high thermal conductivity. For borosilicate, silicon and water standard material properties were used. Only temperature dependent absorption of water in the IR at the wavelength was additionally incorporated into the model [40]. As heating source a Gaussian laser profile was assumed with a beam waist of 30 µm.
As boundary conditions the bottom of the silicon was set to a base temperature and the top of the capillary thermally insulated since heat conduction of the air above is very low. Individual simulation runs were performed for base temperatures in the range of T=5-65 °C. ΔT was extracted by line averaging in the water region over capillary height and subtracting temperatures at the inner and the outer boundary. It can be observed that ΔT decreases with increasing base temperature (Figure 13 b). The main reason is found in the decreasing infrared-absorption coefficient of water with increasing temperature. The result of the numerical evaluation is in good agreement with our measurements of ΔT which was obtained using the temperature sensitive fluorescence of the dye (2',7'-Bis-(2-Carboxyethyl)-5-(and-6)-Carboxyfluorescein) (BCECF). Additionally we probed the effect of variations in capillary thickness (±10%, as stated by the manufacturer). The resulting shift in ΔT is of the order of ±6.3% and was assumed as statistical error for our experiments.

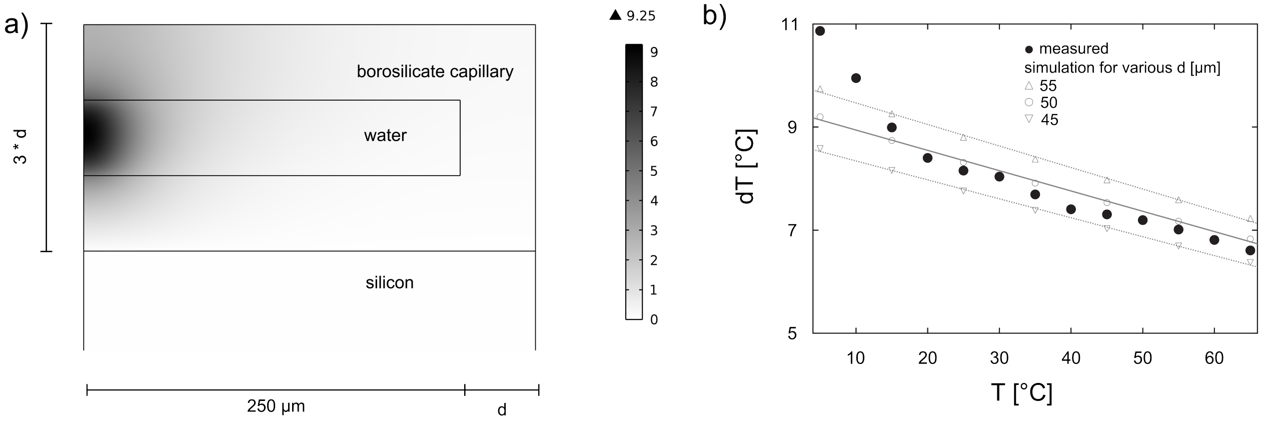

Figure 13: a) Finite Element Simulation for a capillary filled with water which is heated by an infrared (IR)-laser. In this example the temperature at the bottom of the silicon wafer was set to T = 25 °C and the resulting shift by line averaging is ΔT ∼8 °C. b) Since the IR absorbance of water is decreasing with increasing temperature [40], the extracted values for ΔT decrease with increasing base temperature T. Experimental results are in good agreement with our simulation results. The numerical evaluation was also performed for varying capillary thickness *d* of 45, 50 and 55 µm (variation stated by the supplier). A variation of capillary thickness by 10% entails a variation of ΔT of ±6.3% around the mean temperature. This value is used as statistical error of our experiments.

References

[1] Hoyer, W. *et al.* Dependence of α-synuclein aggregate morphology on solution conditions. *J Mol Biol* **322**, 383–393 (2002).

[2] Pinotsi, D. *et al.* Direct observation of heterogeneous amyloid fibril growth kinetics via two-color super-resolution microscopy. *Nano Lett* **14**, 339–345 (2014).

[3] Guilliams, T. *et al.* Nanobodies raised against monomeric α-synuclein distinguish between fibrils at different maturation stages. *J Mol Biol* **425**, 2397–2411 (2013).

[4] Lorenzen, N. *et al.* The role of stable α-synuclein oligomers in the molecular events underlying amyloid formation. *J Am Chem Soc* **136**, 3859–3868 (2014).

[5] Buell, A. K. *et al.* Solution conditions determine the relative importance of nucleation and growth processes in α-synuclein aggregation. *Proc Natl Acad Sci U S A* **111(21)**, 7671–7676 (2014).

[6] Galvagnion, C. *et al.* Lipid vesicles trigger α-synuclein aggregation by stimulating primary nucleation. *Nat Chem Biol* **11**, 229–234 (2015).

[7] Campioni, S. *et al.* The presence of an air-water interface affects formation and elongation of alpha-synuclein fibrils. *J Am Chem Soc* **136(7)**, 2866–2875 (2014).

[8] Buell, A. K. *et al.* Electrostatic effects in filamentous protein aggregation. *Biophys J* **104**, 1116–1126 (2013).

[9] Shammas, S. L. *et al.* Perturbation of the stability of amyloid fibrils through alteration of electrostatic interactions. *Biophys J* **100**, 2783–2791 (2011).

[10] Duhr, S. & Braun, D. Why molecules move along a temperature gradient. *Proc Natl Acad Sci U S A* **103**, 19678–19682 (2006).

[11] Reichl, M., Herzog, M., Götz, A. & Braun, D. Why charged molecules move across a temperature gradient: the role of electric fields. *Phys Rev Lett* **112**, 198101 (2014).

[12] Keil, L. A setup for RNA melting curves and label free thermophoresis: Bachelor thesis (2011).

[13] Mittag, J. J., Milani, S., Walsh, D. M., Rädler, J. O. & McManus, J. J. Simultaneous measurement of a range of particle sizes during Aβ1-42 fibrillogenesis quantified using fluorescence correlation spectroscopy. *Biochem Biophys Res Commun* **448**, 195–199 (2014).

[14] Broersma, S. Rotational diffusion constant of a cylindrical particle. *J. Chem. Phys.* **32**, 1626–1631 (1960).

[15] de la Torre, J. G. G. & Bloomfield, V. A. Hydrodynamic properties of complex, rigid, biological macromolecules: theory and applications. *Q Rev Biophys* **14**, 81–139 (1981).

[16] Herling, T. W. *et al.* Integration and characterization of solid wall electrodes in microfluidic devices fabricated in a single photolithography step. *Applied physics letters* **102**, 184102 (2013).

[17] Henry, D. C. The cataphoresis of suspended particles. Part I. The equation of cataphoresis. *Proc R Soc Lond A* **133**, 106–129 (1931).

[18] Hunter, R. J. *Zeta Potential in Colloid Science: Principles and Applications* (Academic Press, 1981).

[19] Ohshima, H. J. A simple expression for Henry's function for the retardation effect in electrophoresis of spherical colloidal particles. *J. Colloid Interf. Sc.* **168**, 269–271 (1994).

[20] Booth, F. The cataphoresis of spherical, solid non-conducting particles in a symmetrical electrolyte. *Proc R Soc Lond A* **203**, 514–533 (1950).

[21] Klein, S. D. & Bates, R. G. Conductance of tris(hydroxymethyl)-aminomethane hydrochloride (TrisHCl) in water at 25 and 37°C. *J Sol Chem* **9(4)**, 289–292 (1980).

[22] Quist, A. S. & Marshall, W. L. Assignment of limiting equivalent conductances for single ions to 400°C. *J. Phys. Chem.* **69(9)**, 2984–2987 (1965).

[23] Ohshima, H., Healy, T. W. & White, L. R. Accurate analytic expressions for the surface charge density/surface potential relationship and double-layer potential distribution for a spherical colloidal particle. *J. Colloid Interf. Sc.* **90(1)**, 17–26 (1982).

[24] Manning, G. S. Limiting laws and counterion condensation in polyelectrolyte solutions. V. Further development of the chemical model. *Biophys Chem* **9**, 65–70 (1978).

[25] Dhont, J. K. G. & Briels, W. J. *Rod-like Brownian Particles in Shear Flow* (WILEY-VCH Verlag Berlin GmbH, 2004).

[26] Tannous, C. Orientation control of rodlike objects by flow. *arXiv:0905.0091v3* (2009).

[27] Grossman, P. D. & Soane, D. S. Orientation effects on the electrophoretic mobility of rod-shaped molecules in free solution. *Anal Chem* **62**, 1592–1596 (1990).

[28] Newman, J. & Swinney, H. L. Length and dipole moment of tmv by laser signal-averaging transient electric birefringence. *Biopolymers* **15**, 301–315 (1976).

[29] Fixman, M. Charged macromolecules in external fields. 2. preliminary remarks on the cylinder. *Macromolecules* **13**, 711–716 (1980).

[30] Rogers, S. S. *et al.* Electric birefringence study of an amyloid fibril system: the short end of the length distribution. *Eur Phys J E Soft Matter* **18**, 207–217 (2005).

[31] O'Konski, C. T., Yoshioka, K. & Orttung, W. H. Electric properties of macromolecules. IV. Determination of electric and optical parameters from saturation of electric birefringence in solutions. *J. Phys. Chem.* **63(10)**, 1558–1565 (1959).

[32] Ohshima, H. Henry's function for electrophoresis of a cylindrical colloidal particle. *J. Colloid Interf. Sc.* **180**, 299–301 (1996).

[33] Dhont, J. K. G., Wiegand, S., Duhr, S. & Braun, D. Thermodiffusion of charged colloids: single-particle diffusion. *Langmuir* **23**, 1674–1683 (2007).

[34] Reichl, M., Herzog, M., Greiss, F., Wolff, M. & Braun, D. Understanding the similarity in thermophoresis between single- and double-stranded DNA or RNA. *Phys. Rev. E* **91**, 062709 (2015).

[35] Guthrie Jr., G., Wilson, J. N. & Schomaker, V. Theory of the thermal diffusion of electrolytes in a Clusius column. *J. Chem. Phys.* **17**, 310 (1949).

[36] Eslahian, K. A., Majee, A., Maskos, M. & Würger, A. Specific salt effects on thermophoresis of charged colloids. *Soft Matter* **10**, 1931 (2014).

[37] Takeyama, N. & Nakashima, K. Proportionality of intrinsic heat of transport to standard entropy of hydration for aqueous ions. *J. Sol. Chem.* **17(4)**, 305–325 (1988).

[38] Iacopini, S., Rusconi, R. & Piazza, R. The "macromolecular tourist": universal temperature dependence of thermal diffusion in aqueous colloidal suspensions. *Eur Phys J E Soft Matter* **19**, 59–67 (2006).

[39] Römer, F., Wang, Z., Wiegand, S. & Bresme, F. Alkali halide solutions under thermal gradients: Soret coefficients and heat transfer mechanisms. *J Phys Chem B* **117**, 8209–8222 (2013).

[40] Goldstein, R. & Penner, S. The near-infrared absorption of liquid water at temperatures between 27 and 209°C. *Journal of Quantitative Spectroscopy and Radiative Transfer* **4(3)**, 441–451 (1964).
